# The phosphatase PPM1F, a negative regulator of integrin activity, is essential for embryonic development and controls tumor cell invasion

**DOI:** 10.1186/s12915-025-02254-3

**Published:** 2025-06-19

**Authors:** Tanja M. Grimm, Nina I. Dierdorf, Marleen Herbinger, Sarah Baumgärtner, Erik Sontowski, Christoph Paone, Timo Baade, Christof R. Hauck

**Affiliations:** 1 Lehrstuhl Zellbiologie, Fachbereich Biologie, Maildrop 621, Universität Konstanz, Universitätsstrasse 10, Konstanz, Germany; 2 Konstanz Research School Chemical Biology, Universität Konstanz, Konstanz, Germany

**Keywords:** Cell adhesion, FilaminA, Integrin activity, Protein phosphatase, PPM1F, Talin, Threonine phosphorylation, Knock-out mouse, Developmental defects, Cancer cell invasion

## Abstract

**Background:**

The Mn^2+^/Mg^2+^-dependent Ser/Thr phosphatase PPM1F was identified to control integrin activity. Furthermore, PPM1F regulates several protein kinases known to be involved in organizing the cytoskeleton and other cellular functions. Therefore, PPM1F appears critical for a multitude of physiological processes.

**Results:**

Here, we report the phenotype of *ppm1f* gene disruption in mice. While heterozygous *ppm1f* ± mice are viable and fertile, *ppm1f-/-* mice show severe defects and significant morphological abnormalities in the developing brain and vasculature and abort embryonic development at day E10.5. Isolated* ppm1f-/-* MEFs or PPM1F-depleted human neuro-epithelial cells display enhanced integrin-dependent cell adhesion, deregulated PAK phosphorylation, and perturbed cell migration. These phenotypes were reversed by re-expression of the wildtype enzyme, but not the phosphatase-inactive PPM1F. In different human tumor cell types, PPM1F expression levels directly correlated with invasive potential, while deletion of PPM1F abrogates tissue invasion.

**Conclusions:**

These results highlight the non-redundant role of this enzyme in integrin and PAK regulation and identify PPM1F as a promising target to limit tumor metastasis.

**Supplementary Information:**

The online version contains supplementary material available at 10.1186/s12915-025-02254-3.

## Background

Integrins are specialized cell adhesion molecules in metazoa, which perform critical functions during tissue formation and maintenance [1, 2]. Integrins consist of paired α and β subunits, which can occur on the surface of the cell as a closed heterodimer. In this conformation, integrins do not engage in ligand binding and, accordingly, are referred to as inactive integrins [3–5]. By an extensive conformational change, the large extracellular domains of the α and β subunits can be stretched out resulting in an active integrin with high ligand affinity [6, 7]. This massive conformational change can be initiated either by spatial separation of the cytoplasmic tails of the two subunits from within the cell (inside-out-activation) or can be supported by a ligand binding event, which stabilizes the extended conformation (outside-in activation) [5, 8]. As integrins do not possess intrinsic enzymatic activity, both processes are guided by protein–protein interactions, with binding partners of the integrin β subunit serving as positive (e.g. the scaffolding proteins talin and kindlin) or negative (e.g. the actin cross-linking protein filaminA) regulators of integrin activity [9, 10]. Interestingly, previous studies have indicated that the binding of these scaffolding proteins can be regulated by phosphorylation of the integrin β subunit [11]. Initially, the tyrosine residue contained within the conserved NPxY talin binding motif was found to be phosphorylated in vSrc transformed chicken fibroblasts and has been suggested to interfere with talin binding [12–14]. However, mutagenesis of this tyrosine into a non-phosphorylatable phenylalanine did not reveal a difference in focal contact formation and integrin function in vivo [15]. Most integrin β subunits also contain a conserved threonine motif, which is located in the context of the talin, kindlin, and filaminA binding sites. In integrin β1, mutation of threonine 788 (T788) and T789 severely perturbs integrin function, with phospho-mimicking mutations resulting in enhanced integrin activity and cell adhesion, while a T788A/T789A mutant shows impaired cell adhesion [16, 17]. Similarly, the corresponding residues in integrin β2 (T758-T760), a subunit exclusively expressed in hematopoietic cells, have been shown to be phosphorylated in response to cell stimulation by phorbol esters and T-cell receptor (TCR)-clustering and phosphorylation of the threonine motif is associated with increased integrin activity [18–21]. Structural analyses revealed that these threonine residues form an integral part of the filaminA binding site in different integrin β subunits [22, 23]. Biochemical experiments with synthetic peptides indicate that in contrast to talin, filaminA loses its affinity for the cytoplasmic domain of integrin β2 upon T758 phosphorylation [24]. These findings have revived the idea that the conserved threonine motif serves to regulate integrin activity with a kinase-mediated on-switch favoring talin binding and a phosphatase-promoted off-switch, which facilitates filaminA-integrin association [25]. Indeed, a recent study has identified the protein phosphatase PPM1F as the cellular enzyme, which dephosphorylates the T788/T789 motif in integrin β1 [26]. Thereby, the activity of PPM1F is a pre-requisite for filaminA binding to integrin β1, which secures the closed, inactive conformation of the integrin heterodimer [26]. PPM1F is a member of the protein phosphatase 2C (PP2C) family of Mg^2+^/Mn^2+^-dependent protein phosphatases (PPMs) comprising 16 distinct enzymes in humans [27]. PPM1F overexpression has been reported in various types of human cancer including hepatocellular carcinoma, breast and colon cancer, where PPM1F expression levels correlated with increased invasiveness and metastatic behaviour [28–35]. Initially, PPM1F has been described as Calmodulin-dependent kinase phosphatase (CamKP), since it dephosphorylates and inactivates Calmodulin-dependent kinase II (CamKII) [36]. Furthermore, PPM1F has been also termed POPX2 (partner of PIX 2), as it associates with the Cdc42/Rac1 guanine nucleotide exchange factor PIX and dephosphorylates the p21-activated kinase (PAK) [37]. Via binding to p95PKL, PIX associates with the core focal adhesion protein paxillin [38] providing a potential physical link between PPM1F and integrin-based focal adhesions. Though PPM1F clearly can dephosphorylate the conserved threonine motif in the integrin β subunit in vitro and has direct consequences for integrin activity and integrin function in isolated cells [26], the physiological implication of this phosphatase has not been tested in detail in mammals. Integrins and their key interaction partners, such as talin, kindlin, filaminA, paxillin, and focal adhesion kinase, are all essential for mammals, as genetic deletion of these components leads to abortion of early embryonic development [39–45]. In addition, interfering with integrin activity regulation via targeted mutations in the integrin β subunit cytoplasmic domain has similar consequences [15, 46]. Accordingly, if PPM1F indeed serves a critical function in integrin activity regulation, one would expect severe consequences upon inactivation of the *ppm1f* gene.

Here we report the phenotypic analysis of a *ppm1f*-gene trap knock-out allele in mice and studied the consequences of PPM1F-deficiency in normal and transformed cells. PPM1F shows a wide tissue expression during embryonic development and also in adult mice, but with prominent abundance in the central nervous system and hematopoietic cells. Homozygous *ppm1f*-/- murine embryos die in utero around E10.5 showing vascular leakage and severe malformations of the forebrain. While primary fibroblasts isolated from *ppm1f*-/- embryos and PPM1F-deficient human cell lines exhibit enhanced integrin activity, increased PAK phosphorylation, elevated cell adhesion, and distorted cell migration, complementation with the wildtype enzyme, but not with the inactive PPM1F D360A, fully reverts these phenotypes. In several human cancer cell lines, PPM1F expression was directly linked to tissue invasion, which could be abrogated by PPM1F deletion. Consequently, our data support a prominent contribution of PPM1F in regulating integrin function during mammalian development and a critical function of this integrin phosphatase in tumor cell invasion.

## Results

### PPM1F is expressed in multiple tissues in adult mice with high levels in the brain, lung, endothelium, and the hematopoietic system

**Fig. 1:**
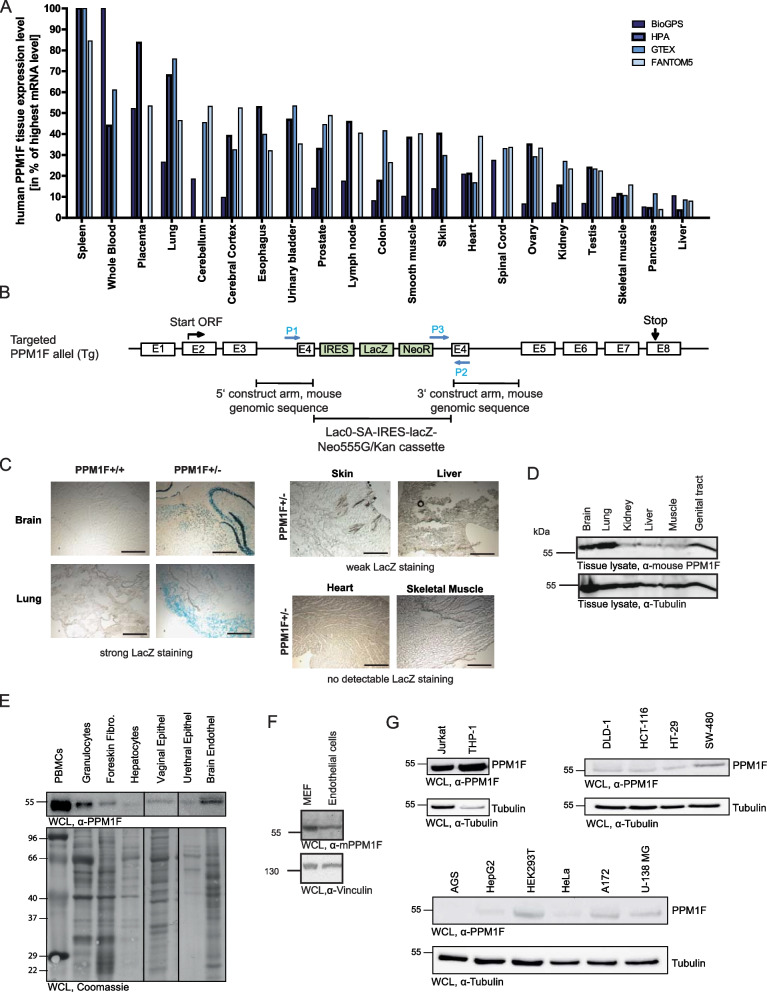
PPM1F is expressed in multiple tissues in the adult with high levels in the brain and hematopoietic system. **A** Schematic comparison of human PPM1F tissue-specific expression levels based on four different transcriptomic studies indicated by differently colored bar graphs (BioGPS, HPA, GTEX, FANTOM5). Mean protein-coding transcripts per million (pTPM)-values for a panel of human tissues analyzed in at least three of the studies were selected and used to calculate the PPM1F expression levels in percentage to the highest PPM1F mRNA level measured within the respective study. Resulting data were plotted from the highest (left) to the lowest (right) values considering all studies. **B** Schematic representation of the targeted *ppm1f* locus. Homologous recombination of a LacZ/neomycin-resistance encoding gene-trap cassette into exon 4 resulted in gene disruption and expression of β-galactosidase under the control of the *ppm1f* gene promoter. Primers used for genotyping are indicated. E: exon number; P1: gene specific primer forward; P2: Gene specific primer reverse; P3: targeted primer forward. **C** Cryosections from the indicated tissues were prepared from adult (> 8-week-old) female wildtype (PPM1F +/+) and PPM1F ± mice. 10 µm thick sections were prepared and stained for β-galactosidase activity; scale bars: 500 µm. **D** 100 mg of indicated tissues from wildtype mice were homogenized in 200 µl lysis buffer, cleared by centrifugation and the supernatant was analyzed by Western blotting with a polyclonal rabbit antiserum directed against murine PPM1F (upper panel) or with a monoclonal anti-tubulin antibody (lower panel). **E** Whole cell lysates of the indicated human primary cells were analyzed by Western blotting with rabbit anti-human PPM1F antibody (upper panel). As a loading control, the membrane was stained with Coomassie (lower panel). **F** Whole cell lysates of murine primary skin endothelial cells and mouse embryo fibroblasts were analyzed by Western blotting with a polyclonal rabbit antiserum directed against murine PPM1F (upper panel) or with monoclonal anti-tubulin antibody (lower panel). **G** Whole cell lysates of the indicated human cell lines were analyzed by Western blotting with rabbit anti-human PPM1F antibody (upper panel) or with monoclonal anti-tubulin antibody (lower panel)

In light of the prominent role of PPM1F for regulating integrins [26], PAK [37], and CamKII [36], we wanted to characterize the physiological relevance of this enzyme in the intact organism in more detail. First, we retrieved human PPM1F tissue expression data from a panel of transcriptomic studies including the Human Protein Atlas transcriptomic study, the Genotype-Tissue Expression project, the Functional Annotation of Mammalian Genome 5 (FANTOM5) project and the BioGPS Gene Atlas, which indicate a wide tissue expression of human PPM1F, with particular high expression levels in brain, lung, and reproductive tract (Fig. 1 A). To verify these high throughput transcript data, we employed mice containing a gene-trap insertion in exon 4 of the *ppm1f* gene resulting not only in disruption of the *ppm1f* gene, but also in lacZ expression under control of the endogenous *ppm1f* promoter (Fig. 1 B) [26]. In a heterozygous *ppm1f* ± background, this allele allowed us to monitor the gene expression pattern of PPM1F in adult mice. LacZ staining showed that the *ppm1f* promoter is active in multiple tissues of adult mice with high expression levels in brain and lung, similar to what has been reported for human tissue (Fig. 1 C). Furthermore, weak LacZ staining was observed in skin and liver sections, while no LacZ staining was seen in heart and skeletal muscle (Fig. 1 C). To determine if promoter activities correlate with the amount of protein, we analysed PPM1F protein levels in various tissues. Western Blotting using a polyclonal antibody raised against murine PPM1F confirmed that brain and lung displayed relative high PPM1F protein levels, medium levels in reproductive tissue, while liver, kidney or skeletal muscle contained relative low amounts of this phosphatase (Fig. 1 D). In isolated human primary cells, PPM1F expression was relatively high in peripheral blood mononuclear cells (PBMCs), granulocytes, and brain endothelial cells, a medium level was seen in foreskin fibroblasts, while low PPM1F levels were detected in isolated hepatocytes, vaginal epithelial cells, and urethral epithelial cells (Fig. 1 E). To confirm the expression of PPM1F in endothelial cells, we isolated primary murine endothelial cells from tail skin by tissue dissociation and cluster of differentiation 31 (CD31)-directed cell sorting. Western Blots of lysates generated from these isolated primary murine endothelial cells showed a clear positive signal for PPM1F (Fig. 1 F). The expression pattern of PPM1F in human primary cells was also reflected by the expression of this protein phosphatase in different human cell lines with high levels observed in the monocytic THP-1 and lymphoid Jurkat cell lines (Fig. 1 G). Medium expression levels of PPM1F were observed in various colon carcinoma lines (DLD-1, HCT-116, HT-29, SW480), embryonic kidney cells (HEK293) and in glioblastoma cells (A172, U-138MG), while carcinoma cells derived from liver (HepG2) or genital tract (HeLa) showed low levels and gastric carcinoma cells (AGS cells) had no detectable PPM1F expression (Fig. 1 G). These analyses demonstrate that PPM1F has a wide tissue expression in mammals, but also suggest a particular role for PPM1F in the brain as well as the hematopoietic and immune system.

### PPM1F knock-out embryos show malformation of the central nervous system and the vasculature resulting in early abortion of development

**Fig. 2:**
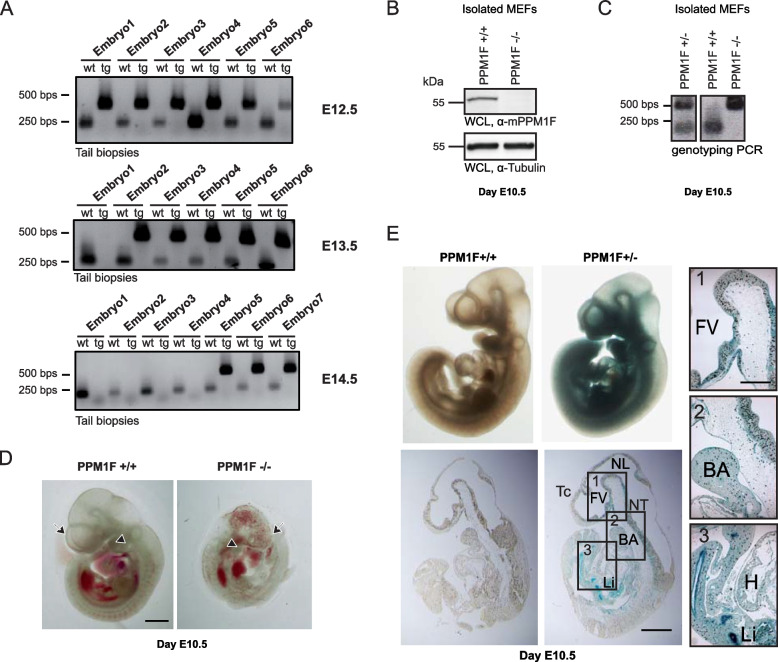
PPM1F knock-out embryos show severe defects, resulting in early abortion of development. **A** PPM1F ± mice were mated and embryos were isolated and genotyped at 12.5, 13.5 and 14.5 days post coitus. **B** WCLs of MEFs isolated at day E10.5 from regularly developed (putative PPM1F +/+ and PPM1F ±) or malformed (putative PPM1F-/-) embryos were probed with polyclonal α-mPPM1F antiserum (upper panel) or monoclonal α-tubulin (lower panel). **C** Genomic DNA was extracted from E10.5 fibroblasts as in (**B**). Genotyping PCR identified WT, heterozygous and homozygous *ppm1f* KO embryos. **D** Isolated *ppm1f*^*−/−*^ embryo at E10.5 (right picture) is smaller in size compared to a wildtype embryo and shows a stunted forebrain and hemorhages (left picture); malformation of the telencephalon (arrow) and branchial archs (arrowhead) in the *ppm1f*^*−/−*^ embryo is indicated; scale bar: 1 mm. **D** Whole-mount X-gal staining and sagittal sections of representative *ppm1f*^+/+^ (left picture) and *ppm1f*^±^ embryo (right picture) at E10.5 showing strong β-galactosidase expression in the rostral region of the telencephalon, the neural tube, the liver and lung. Tc: telencephalon; FV: forebrain vesicle; NT: neural tube; NL: neural lumen; BA: branchial arch; H: heart; Li: liver; scale bar: 500 μm. Areas marked with numbered boxes are shown enlarged on the right hand side; scale bar enlargement: 150 µm

As regulation of integrin activity is critical for tissue maintenance, but also essential for embryonic development [2, 47], we hypothesized that the genetic deletion of PPM1F should have a prominent phenotype in vivo*.* As observed before, crosses between wildtype (*ppm1f* + */* +) and heterozygous (*ppm1f* ±) mice led to the expected 50:50 ratio of *ppm1f* + */* + and *ppm1f* ± offspring, while crosses between heterozygous *ppm1f* ± animals yielded no homozygous *ppm1f-/-* pups, suggesting that PPM1F-deficient embryos die in utero [26]. To determine the time point of abrogation of embryonic development, we performed timed matings and isolated embryos at E10.5, E12.5, E13.5, or E14.5 after conception. Genotypic analysis of isolated embryos showed that *ppm1f-/-* embryos were not present at embryonic day E12.5, E13.5, or E14.5 (Fig. 2A). Amongst the isolated embryos at day E10.5, we could detect ~ 20% (9 out of 50 total) with retarded development. To unambiguously assign a genotype to day E10.5 embryos, we isolated primary murine embryonic fibroblasts (MEFs) from embryos with retarded development (putative ppm1f-/-) and from normally developed embryos to conduct Western blotting with whole-cell lysates and to analyse their genomic DNA. The isolated cells from small, malformed embryos completely lacked PPM1F expression (Fig. 2 B) and harboured two disrupted PPM1F alleles (*ppm1f-/-*), while the regularly developed embryos were either homozygous (*ppm1f* + */* +*)* for the wildtype allele or heterozygous (*ppm1f* ±) (Fig. 2 C). The *ppm1f-/-* embryos at E10.5 were about half the size of the other embryos, displayed malformed forebrain structures, and showed reduced development of the branchial archs (Fig. 2 D). Most of these stunted embryos also had haemorrhagic areas, which were not seen in the regularly developed embryos (Fig. 2 D). LacZ staining of regularly developed day E10.5 *ppm1f* ± embryos revealed that at this point in development the *ppm1f* gene promoter has its highest activity in neuronal tissues of the head and neuronal tube regions, as well as in the heart, branchial arch, and liver (Fig. 2 E). The high expression levels of PPM1F in the brain, branchial arch and vasculature in the regularly developed embryos correlated with the observed malformations of the *ppm1f-/-* embryos. These results underscore the essential role of PPM1F during embryonic development and point to a critical function of this phosphatase during cortex development.

### *Ppm1f-/-* embryos display distorted cell and matrix organization in the forebrain

**Fig. 3:**
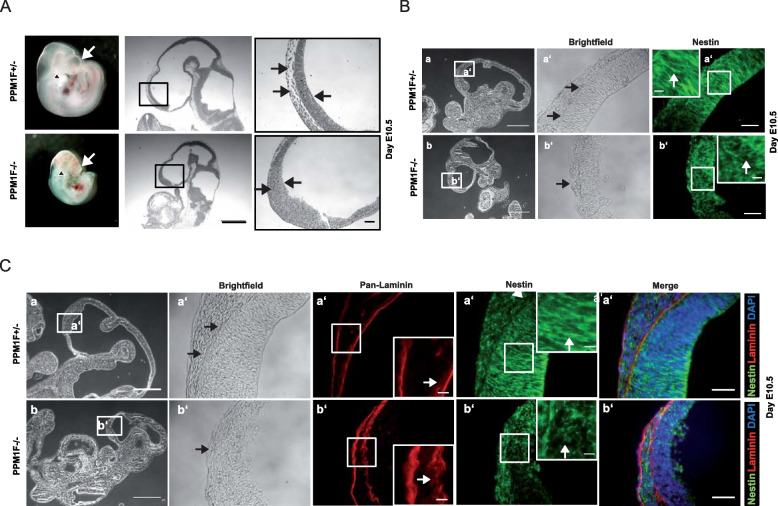
*Ppm1f-/-* embryos display distorted cell and matrix organization in the forebrain. **A** Whole mount *ppm1f*^±^ and *ppm1f*^*−/−*^ embryos at day E10.5 (left panel). *Ppm1f*^*−/−*^ embryos display a stunted telencephalon (white arrow), reduced development of branchial archs (black arrowhead) and bleeding. Sagittal sections of paraffin-embedded embryos were stained with H&E; Middle panel: overview of head region, scale bar 500 µm. Right panel: detailed view of boxed area; normal tissue borders in wildtype embryos and loss of tissue layers in *ppm1f*^*−/−*^ embryos are indicated by black arrows. Scale bar 50 µm. **B** Cryosections of E10.5 *ppm1f*^±^ and *ppm1f*^−/−^ embryos stained for nestin as indicated; scale bars: 500 μm (leftmost column), 50 μm (right columns). Insets show higher magnification of boxed areas; scale bar: 10 µm. While neuroepithelial cells strech and align in dorso-ventral direction in wildtype embryos, the neural progenitor cells within the ventricular zone of *ppm1f*^−/−^ embryos are disoriented (white arrows). **C** Cryosections as in (**B**) were co-stained for laminin (red) and nestin (green). Nuclei were stained by DAPI (blue). Scale bars: 500 μm (leftmost column), 50 μm (right columns). Insets show higher magnification of boxed areas; scale bar: 10 µm. While a straight and narrow layer of laminin is seen in the wildtype, this layer is widened and contorted in *ppm1f*.^−/−^ embryos (white arrows) co-inciding with the disorientation of the nestin-positive neuronal progenitor cells. See also Additional_File1

Given the dramatic defects in the developing cortex, we performed histological analysis of the head region of E10.5 embryos (Fig. 3 A). In contrast to wildtype embryos, *ppm1f -/-* embryos lacked a clear separation between the pia mater and the neuroepithelial cells within the ventricular zone of the telencephalon (Fig. 3 A). Importantly, the formation of the brain cortex is initiated at around day E10.5 by neuroepithelial cells from within the ventricular zone, which by proliferation and migration generate the distinct cortical layers [48]. In wildtype murine embryos, these neuroepithelial cells, which were stained for the neural marker protein nestin, build a parallel and regularly spaced scaffold that terminates in well-defined end feet at the pial surface (Fig. 3 B). However, in PPM1F knockout embryos this defined organization and orientation of neural progenitor cells within the ventricular zone of the caudal telencephalon was lost (Fig. 3 B). Furthermore, laminin staining of the basement membrane indicated a defined and sharp separation of the meningeal epithelium from the ventricular zone in wildtype embryos, while this extracellular matrix structure was contorted and widened in the PPM1F-deficient embryos (Fig. 3 C). As the genetic knock-out of PPM1F had such a severe phenotype on neuronal tissue development, we wondered about brain morphology and function in adult heterozygous mice. Clearly, PPM1F expression in brain tissue of heterozygous *ppm1f* ± mice was only about 50% of wildtype levels, but the size and histological appearance of the brain in these mice did not show obvious abnormalities (see Additional File 1: Fig S1). Thus, a definitive answer to the role of PPM1F in the adult brain has to await the generation of tissue-specific knock-out mice. However, our findings in *ppm1f-/-* embryos illustrate the essential role of this phosphatase in proper cortex formation, where the disorganization of neuronal precursors and their supporting extracellular matrix structures explains the macroscopically observed malformation of the forebrain.

### Increased integrin β1 activity, elevated cell adhesion, and migration defects of *ppm1f-/-* MEFs are reverted by re-expression of wildtype PPM1F

**Fig. 4:**
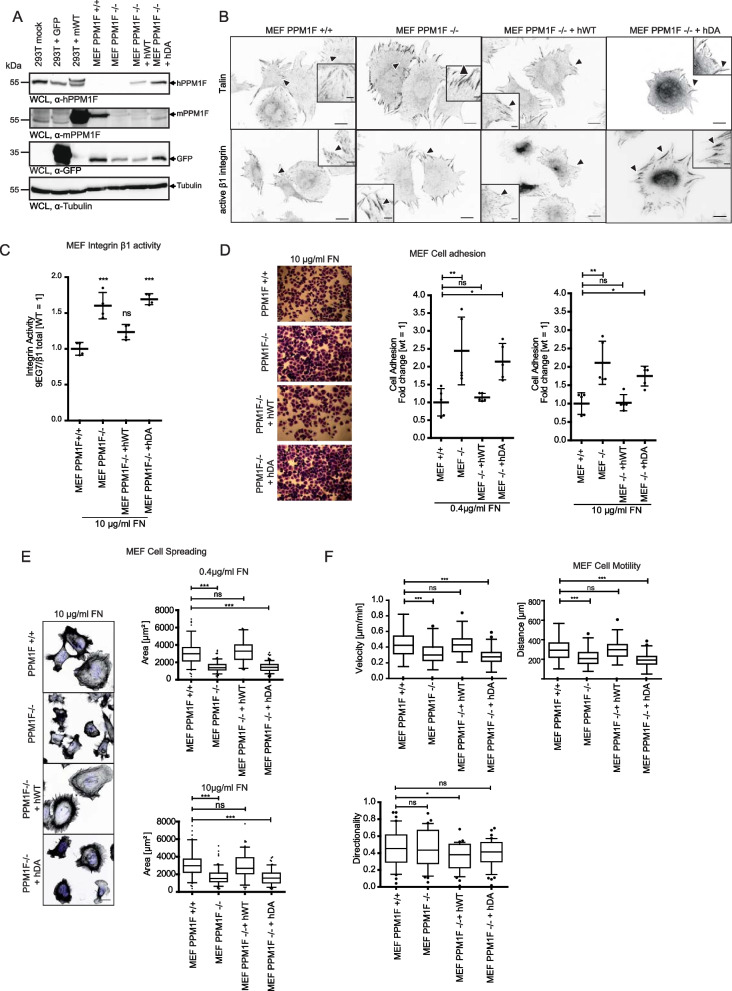
Increased integrin β1 activity, elevated cell adhesion, and migration defects of *ppm1f-/-* MEFs are reverted by re-expression of wildtype PPM1F. **A** PPM1F-/- MEFs were transduced with lentiviral particles encoding human wildtype PPM1F (hWT) or human PPM1F D360 A (hDA) in a bi-cistronic expression cassette with GFP. In addition, PPM1F-/- MEFs and PPM1F +/+ cells were transduced with a lentivirus encoding GFP alone. WCLs of sorted cells were analyzed by Western blotting with the indicated antibodies; as controls, WCLs of 293 T cells transfected with the empty vector (mock), GFP (GFP) or murine PPM1F (mWT) were loaded. **B** MEFs as in (**A**) were seeded onto 1 µg/ml FN_III9-12_ for 2 h. Samples were fixed and stained for talin (upper panel) or the active integrin β1 (lower panel) before analysis by confocal microscopy; scale bar: 20 µm. Insets show higher magnification of boxed areas; scale bar: 5 µm. Arrowheads point to active integrin β1 or talin enrichment. **C** MEFs as in (**A**) were kept in suspension for 45 min and incubated for 15 min with 10 µg/ml FN_III9-12_ (FN). Samples were stained for total (Hmb1-1) or active β1 integrin (9EG7) and analyzed by flow cytometry, ≥ 10 000 counts. The mean fluorescence intensity (MFI) ratio of active to total β1 integrin was calculated and normalized to the wildtype sample (= 1). Scatter blots represent mean ± SEM of 4 independent experiments; statistics was performed using one-way ANOVA and Bonferroni post-hoc test (p*** < 0.001, ns = not significant). **D** MEFs were seeded in triplicates onto fibronectin-coated wells for 60 min and cell adhesion was quantified. Representative pictures from cells seeded on 10 µg/ml FN (left panel); scale bar: 150 µm. Scatter blots represent mean ± SEM of 5 independent experiments performed in technical triplicates each. Values were normalized to MEF wildtype cells (= 1). Statistics was performed using one-way ANOVA, followed by Bonferroni post-hoc test (**p < 0.01, *p < 0.05, ns = not significant). **E** MEFs were seeded onto indicated fibronectin concentrations for 45 min, fixed and stained with DAPI and Phalloidin-Cy5. Samples were imaged using confocal microscopy. Representative images from cells seeded onto 10 µg/ml FN are shown; scale bar: 10 µm (left panel). Quantification of cell spreading. Boxes and whiskers indicate median with 95% confidence intervals from 2 independent experiments; *n* ≥ 90 cells. Statistics was performed using one-way ANOVA, followed by Bonferroni post-hoc test (***p < 0.001, ns = not significant) (right panel). **F** Serum starved MEFs were stimulated by addition of 10% FCS and cell migration was monitored every 30 min for 12 h using time-lapse microscopy. Cell tracks were evaluated for velocity, covered distance and directionality. Boxes and whiskers indicate median with 95% confidence intervals from 2 independent experiments (*n* = 30); Statistics was performed as in (**E**); ***p < 0.001, * *p* < 0.05, ns = not significant. See also Additional_File2

Though PPM1F-deficiency could not be further analysed in adult mice, we used isolated primary fibroblasts from wildtype (WT) and *ppm1f-/-* murine embryos. These cells exhibit increased integrin activity accompanied by marked talin recruitment resulting in exaggerated cell adhesion and in migration defects [26]. To unambiguously assign these phenotypes to PPM1F and identify potential secondary effects, we complemented the PPM1F-deficient primary murine embryonic fibroblasts (MEF PPM1F-/- cells) with either the active human enzyme (MEF PPM1F-/- + hWT) or with the phosphatase-dead mutant human PPM1F D360A (MEF PPM1F-/- + hDA) (Fig. 4 A). A monoclonal antibody directed against human PPM1F detected the enzyme in human 293T cells and comparable levels of the human enzyme were expressed in the MEF PPM1F-/- cells complemented with hWT and hDA (Fig. 4 A). Though this monoclonal antibody detected the overexpressed murine homologue in 293T cells, it did not detect the endogenous levels of this enzyme in wildtype murine fibroblasts (Fig. 4 A). Therefore, we raised a polyclonal antiserum against mouse PPM1F in rabbits, which detected the slightly larger murine protein in wildtype MEF and in 293T cells transfected with an expression construct encoding murine PPM1F (Fig. 4 A and Additional File2: Fig. S2 A). Clearly, murine PPM1F was completely absent from MEF PPM1F-/- cells and from the reconstituted MEF lines, which instead expressed human wildtype PPM1F or the inactive PPM1F D360A (Fig. 4 A). As observed before, PPM1F-/- MEFs seeded on fibronectin displayed elevated levels of active integrin β1, which accumulated together with talin at peripheral focal adhesion sites (Fig. 4 B). Most importantly, re-expression of wildtype human PPM1F in PPM1F-/- cells (MEF PPM1F-/- + hWT) completely reverted this phenotype and the PPM1F re-expressing cells showed the regular pattern of active integrin and talin with small peripheral focal adhesions (Fig. 4 B). Interestingly, expression of inactive PPM1F D360A exaggerated the phenotype of MEF PPM1F-/- cells and led to extraordinary large assemblies of active integrin β1 (Fig. 4 B). In addition, the subcellular distribution of talin was altered in PPM1F D360A re-expressing PPM1F-/- cells, with slightly elevated levels of talin in the perinuclear cytoplasm (Fig. 4 B). Importantly, the re-expression of wildtype or inactive PPM1F did not alter integrin surface levels and did not modify the expression levels of major adhesome proteins (see Additional File2: Fig. S2B and C). Compared to wildtype MEFs, the PPM1F-/- cells showed elevated levels of the integrin α_V_ subunit on the cell surface, which remained stable upon re-expression of PPM1F in MEF PPM1F-/- + hWT as well as MEF PPM1F-/- + hDA cells (see Additional File2: Fig. S2 C and D).

In line with the increased talin recruitment, MEF PPM1F-/- also showed elevated integrin activity compared to wildtype cells (Fig. 4 C). This directly translated into stronger adhesiveness of the MEF PPM1F-/- cells on low (0.4 µg/ml) as well as high (10 µg/ml) concentrations of the integrin ligand fibronectin (Fig. 4 D). Again, both these phenotypes were reverted upon re-expression of wildtype human PPM1F, but not by re-introduction of the inactive enzyme (Fig. 4 C, D). As the histological analysis of PPM1F-deficient embryos revealed altered cell morphology and aberrant tissue organization, we wondered, whether the enhanced integrin-mediated cell adhesion of MEF PPM1F-/- cells would influence initial cell spreading and, more importantly, would corrupt integrin-dependent cell motility. Indeed, we could observe a strong retardation of initial spreading by MEF PPM1F-/- cells and MEF PPM1F-/- hDA cells, while spreading of PPM1F-/- hWT cells was indistinguishable from wildtype MEF (Fig. 4 E). Not surprisingly, the MEF PPM1F-/- cells with their elevated integrin activity and their impaired initial cell spreading showed reduced cell migration velocity and migrated a significantly shorter distance in the same time frame as the wildtype cells (Fig. 4 F). This defect was rescued completely by re-expression of active PPM1F, but not by PPM1F D360 A (Fig. 4 F). In summary, our data confirm the severe effect of PPM1F deletion on integrin activity in primary cells and clearly demonstrate that the PPM1F knock-out phenotype is a direct consequence of the lack of PPM1F activity. Our findings of a gain-of-function with regard to integrin activity in PPM1F-/- cells also corroborate the role of PPM1F as a prominent negative regulator of integrin β1 function.

### Knock-down of PPM1F or filaminA in neuro-epithelial cells increases cell adhesion and compromises haptotaxis

**Fig. 5:**
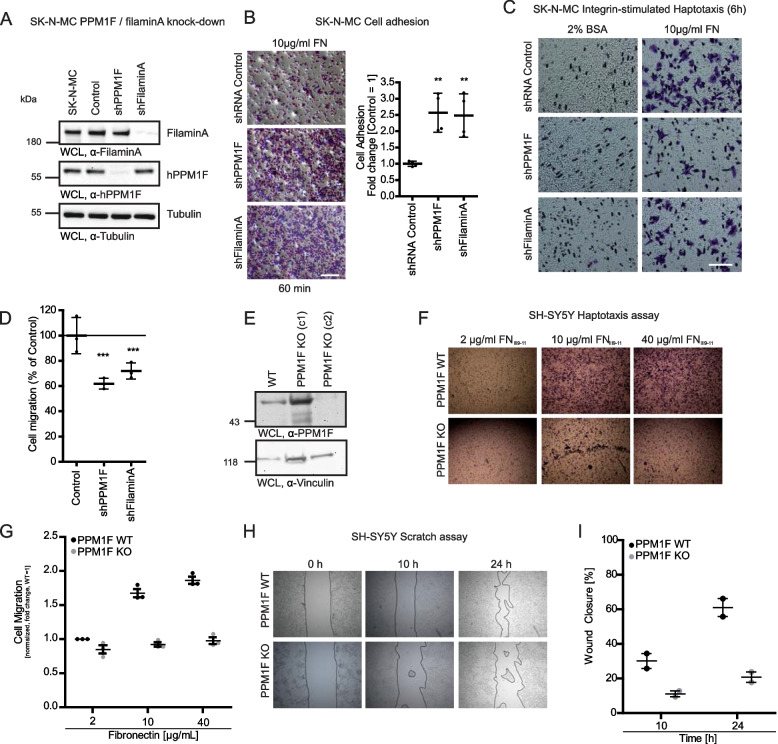
Knock-down of PPM1F or filaminA in neuro-epithelial cells increases cell adhesion and compromises haptotaxis. **A** SK-N-MC wildtype cells were transduced with lentiviral particles harboring shRNA against human filaminA, human PPM1F or scrambled shRNA as control and puromycin selection was performed. WCLs were prepared and subjected to Western blotting with indicated antibodies. **B** SK-N-MC cells from (**A**) were seeded in triplicates onto fibronectin-coated wells for 60 min and cell adhesion was quantified. Representative pictures from cells seeded on 10 µg/ml FN (left panel); scale bar: 150 µm. Graph on the right depicts values of 4 independent experiments performed in technical triplicates. Values were normalized to SK-N-MC control cells (= 1). Lines and whiskers indicate means ± SEM. Statistics was performed using one-way ANOVA, followed by Bonferroni post-hoc test (**p < 0.01). **C** Haptotaxis motility assays were performed by seeding serum-starved SK-N-MC cell lines on top of a Boyden chamber membrane coated on the lower side with 10 µg/ml FN or 2% BSA as control. Cells, which had migrated to the lower side of the membrane, were quantified after 6 h by crystal violet staining and counting of five fields/chamber under the light microscope (40 × objective). Representative images of the lower side of the membrane are shown; scale bar: 50 µm. **D** Quantification of haptotaxis motility assays in (**C**). Depicted are numbers of migrated cells ± SEM from 10 µg/ml FN treated samples from three independent experiments performed in technical triplicates. Lines and whiskers indicate means ± SEM. Statistics was performed using one-way ANOVA, followed by Bonferroni post-hoc test (***p < 0.001). **E** CRISPR/Cas-mediated knock-out of PPM1F in SH-SY5Y cells. WCLs from wildtype (WT) and PPM1F KO SH-SY5Y cells were analyzed by Western blotting with α-human PPM1F (upper panel). α-Vinculin antibody was used as loading control (lower panel). **F**, **G** Haptotaxis motility assays with SH-SY5Y cells from (**E**) with Boyden chambers coated with 2, 10, or 40 µg/ml FN performed as in (C). Representative images of the lower side of the membrane are shown. Graph shows the quantification of a representative experiment performed in technical triplicates (**F**). **H**, **I** Wound healing assay with SH-SY5Y cells from (**E**). Wound closure was monitored over 24 h and representative images are shown. Graph shows the quantification of wound closure from a representative experiment performed in technical triplicates. See also Additional_File 3

The in vivo analysis of PPM1F not only underscored the prominent expression of this protein in neuronal tissues, but also illustrated the severe manifestations of PPM1F deficiency in the developing brain. Interestingly, a similar misorganization of the prefrontal cortex has been observed in filaminA knock-out mouse embryos [43] and filaminA can reduce integrin activity in fibroblasts upon PPM1F-mediated dephosphorylation of the integrin β1 subunit [26]. To investigate, if PPM1F and filaminA together modulate integrin-based cell adhesion in neuronal cells, we produced a stable knock-down of either protein in human SK-N-MC neuro-epithelial cells (Fig. 5A, see Additional File 3: Fig. S3 A and B). The depletion of PPM1F or filaminA did not alter expression levels of core focal adhesion proteins and also integrin surface levels were unchanged (see Additional File: Fig. S3B and C). However, silencing of either PPM1F or filaminA led to a gain-of-function with regard to integrin-based cell attachment to fibronectin (Fig. 5B). In comparison to cells treated with a non-targeting control shRNA, the depletion of filaminA as well as the depletion of PPM1F resulted in a strong elevation (~ twofold) in cell adhesion, indicating that PPM1F as well as filaminA function as negative regulators of integrin β1 activity in these neuronal cells (Fig. 5 B). In line with the increased cell adhesion, PPM1F-depleted as well as filaminA-depleted cells showed reduced migration towards an extracellular integrin ligand in haptotaxis assays (Fig. 5 C and D). To verify the severe migration defect of neuroblastoma cells upon lack of PPM1F, we created a second, independent PPM1F-deficient cell line by CRISPR/Cas9-mediated disruption of the *PPM1F* gene in the SH-SY5Y neuroblastoma cell line. Transduction with a Cas9/sgRNA-PPM1F-encoding lentivirus followed by puromycin selection resulted in SH-SY5Y cells that lacked PPM1F expression (Fig. 5 E). Both, in fibronectin-directed Boyden-chamber haptotaxis assays as well as in wound healing assays on a collagen-coated surface, the PPM1F-deficient cells showed a strong impairment of haptotaxis migration and 2D-wound closure (Fig. 5 F – I).

These data extent previous findings of a PPM1F-controlled phospho-switch in integrin β1 [26]. The similar consequences of PPM1F- or filaminA-deficiency for the migratory potential of neuroepithelial cells provide a mechanistic explanation for the increased accumulation of neuronal progenitor cells at the ventricular zone in both *ppm1f-/-* and *filaminA-/-* embryos.

### PPM1F contributes to the invasive phenotype of tumor cells

**Fig. 6:**
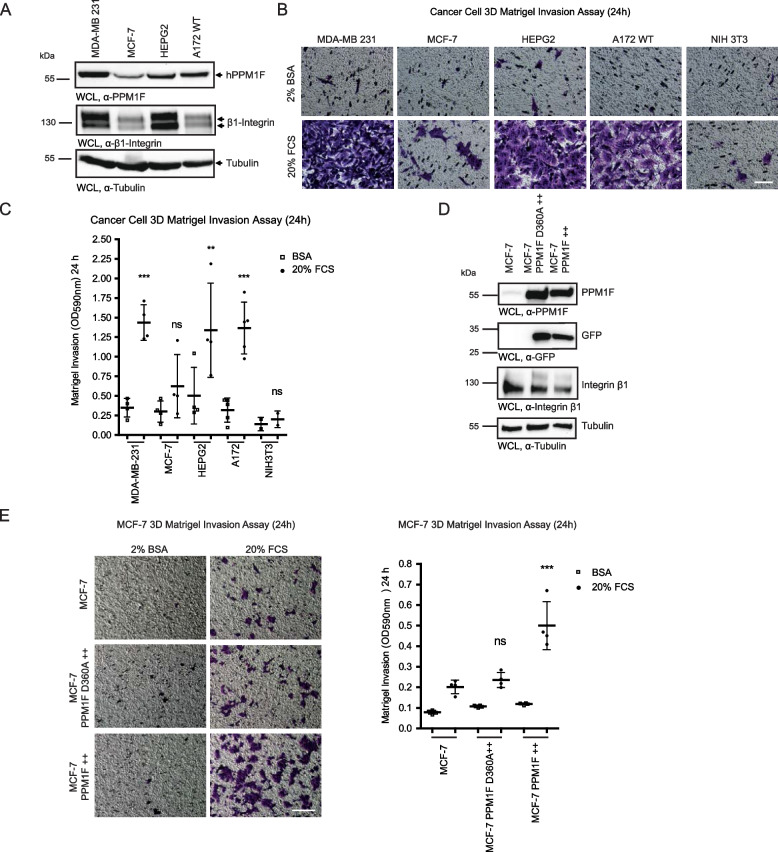
PPM1F contributes to the invasive phenotype of tumor cells. **A** WCLs from indicated cancer cell lines were analyzed by Western blotting with α-human PPM1F or α-integrin β1 antibodies. α-Tubulin antibody was used as loading control. **B**, **C** Indicated serum-starved cancer cells were seeded on top of a Matrigel basement membrane (30 µg/100 µl) in Boyden chamber cell invasion assays using 20% FCS as stimulus or 2% BSA to evaluate random invasion activity. NIH3 T3 cells served as non-invasive control cells. Representative pictures of the lower porous membrane surface (20x) are shown in (B); scale bar: 50 µm. Crystal violet-stained cells can be distinguished from the 8 µm membrane pores. Cells were evaluated for invasion after 24 h by dye elution with 10% acetic acid and absorbance measurement at 590 nm. Graph in (**C**) shows quantified means ± SEM from three independent experiments. Statistics was performed using one-way ANOVA and Bonferroni post-hoc test (p*** < 0.001, p** < 0.01, ns = not significant). **D** MCF-7 cells were stably transduced with lentiviral particles harboring a bicistronic GFP and hPPM1F wildtype or hPPM1F D360 A expression cassette and single-cell sorted via flow cytometry for GFP positive cells to obtain a mixed population of PPM1F-overexpressing MCF-7 cells (PPM1F + + and PPM1F D360 A + +). WCL of the wildtype and modified cell lines were analyzed by Western blotting with indicated antibodies. α-tubulin antibody (lowest panel) served as loading control. **E** Serum-starved cells from (**D**) were seeded on top of a Matrigel base (30 µg/100 µl) in Boyden chambers. Cell invasion was stimulated by addition of 20% FCS or 2% BSA to the lower chamber. Representative pictures of the lower porous membrane surface (20x) are shown; scale bar: 50 µm. Crystal violet-stained cells can be distinguished from the 8 µm membrane pores. Invasion was quantified by dye elution. Graph (right) shows means ± SEM from four independent experiments performed in triplicate. Statistics as in (**C**)

Given that PPM1F controls integrin activity and matrix-dependent cell migration, it is not surprising that increased PPM1F expression has been connected to the invasive phenotype of carcinoma cells [28–35, 49–51]. As reported before, PPM1F expression levels were higher in MDA-MB 231 breast carcinoma cells and HepG2 hepatocellular carcinoma cells compared to epithelial-like MCF-7 cells [28, 29, 52] (Fig. 6 A). However, integrin β1 levels were also increased in the two carcinoma cell lines compared to MCF-7 cells, which could also explain their pronounced invasive phenotype [53–56] (Fig. 6 A). Therefore, we also included A172 glioblastoma cells in our study, which show high PPM1F expression, while integrin β1 levels are similar to MCF-7 cells (Fig. 6 A). Importantly, MCF-7 cells exhibited a low invasive potential in 3D Matrigel invasion assays comparable to non-transformed NIH3 T3 fibroblasts (Fig. 6 B and C). In contrast, Hep G2, MDA-MB 231, and A172 cells were highly invasive (Fig. 6 B and C) correlating with the elevated PPM1F expression levels in these cells, but not with integrin β1 levels (Fig. 6 A). To directly test the contribution of PPM1F to the invasive phenotype of these cells, we stably overexpressed either the active phosphatase or the inactive enzyme in non-invasive MCF-7 cells (Fig. 6 D). Overexpression of wildtype PPM1F produced a strong gain of function with regard to matrix invasion by the transduced MCF-7 cells, allowing these cells to penetrate a matrigel barrier in vitro (Fig. 6 E). In contrast, MCF-7 cells expressing PPM1F D360A showed the same low invasive behaviour as the parent MCF-7 cell line (Fig. 6 E). Together, these results strongly support the notion that PPM1F overexpression can contribute to the metastatic phenotype of carcinoma cells.

### Genetic deletion of PPM1F in tumor cells diminishes matrix invasion

**Fig. 7:**
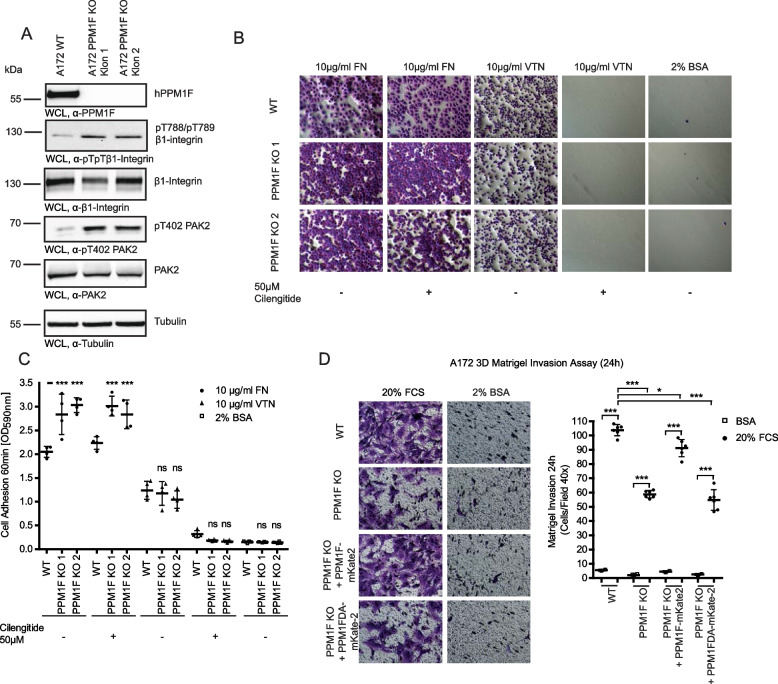
Genetic deletion of PPM1F in tumor cells diminishes matrix invasion and integrin phosphorylation. **A** WCLs from A172 wildtype cells and two clonal PPM1F KO cell lines (1 and 2) were analyzed by Western blotting using the indicated antibodies. α-Tubulin antibody was used as loading control. **B** Serum starved A172 wildtype cells and PPM1F KO cell lines (clone 1 and clone 2) were seeded in triplicate onto fibronectin-, vitronectin-, or 2% BSA-coated wells for 60 min either in presence of 50 µM cilengitide or DMSO as control. Wells were washed and adherent cells were stained with crystal violet. Representative pictures are shown; scale bar: 150 µm. **C** Adherent crystal violett stained cells from (**B**) were quantified by dye elution. Graph depicts individual values as well as mean ± SEM of 4 independent experiments performed in technical triplicates. Statistics was performed using one-way ANOVA, followed by Bonferroni post-hoc test (***p < 0.001; **p < 0.01; p* < 0.05; ns = not significant) and shown for the PPM1F knock-out clones in relation to the A172 wildtype cells. **D** Serum-starved cells as in (**C**) were seeded on top of a Matrigel base (30 µg/100 µl) in Boyden chambers and cell invasion was stimulated by addition of 20% FCS or 2% BSA to the lower chamber. Cells were evaluated for invasion after 24 h and representative pictures of the lower porous membrane surface (20x) are shown; scale bar: 50 µm. Crystal violet-stained cells can be distinguished from the 8 µm membrane pores (left). Invasion assays were quantified by dye elution. Graph depicts individual values as well as means ± SEM from four independent experiments performed in triplicate. Statistics as in (**C**). See also Additional_File4 and Additional_File5

To investigate, if high PPM1F levels sustain the invasive phenotype of transformed cells, we chose A172 glioblastoma cells and generated clonal PPM1F-knock-out A172 cell lines (PPM1F KO) by CRISPR/Cas9-mediated gene disruption [26]. Two independent clonal lines of A172 PPM1F KO (KO1 and KO2) with complete absence of PPM1F expression were analyzed by Western Blotting for increased phosphorylation of PPM1F target proteins (Fig. 7 A). Indeed, both PPM1F KO clones showed elevated levels of integrin β1 T788/T789 and PAK2 T402 phosphorylation (Fig. 7 A). Though the used polyclonal antibody selectively binds to recombinant, phosphorylated pT788/pT789 integrin β1 (Additional File 4: Fig. S4 A), it can not be ruled out that this antibody detects unrelated, doubly phosphorylated proteins in whole cell lysates as suggested by others [57]. Also in our experiments, we have observed that different batches of this commercial antibody preparation can recognize in whole cell lysates (WCLs) proteins with various apparent molecular weights (compare e.g. the WCL samples in Fig. 7A with WCL samples in Additional File4: Fig. S4D). To ascertain that the detection by this antibody is focussed on integrin β1, we first immunoprecipitated integrin β1 using a mouse monoclonal integrin-specific antibody (P5D2) from lysates of A172 wildtype and A172 PPM1F KO cells. Control immunoprecipitation with an isotype matched control IgG did not yield an integrin-reactive band, while clone P5D2 precipitated equal amounts of integrin β1 from each lysate (Additional File4: Fig. S4 A, B, D). Probing immunoprecipitates generated with anti-integrin β1 antibody P5D2 with the polyclonal antibody against phosphorylated pT788/pT789 integrin β1 detected a band of ~ 120–130 kDa. Importantly, A172 PPM1F KO cells consistently showed a higher reactivity than A172 WT cells (Additional File 4: Fig. S4B, D). Similarly, immunoprecipitates generated with a 1:1 mixture of two different rat monoclonal anti-integrin β1 antibodies (clone 9EG7, recognizing the active integrin β1- and clone AIIB2, recognizing integrin β1 irrespective of the activation status) also yielded bands reactive with the polyclonal pT788/pT789 integrin β1 antibody with elevated phosphorylation in the A172 PPM1F KO cells (Additional File 4: Fig. S4B, D). The membrane was stripped and reprobed with a rabbit monoclonal integrin β1 antibody (clone D2E5) to confirm similar amounts of integrin β1 in the precipitates (Additional File 4: Fig. S4B, D). As an additional approach, surface proteins of A172 WT and A172 PPM1F KO cells were biotinylated and upon cell lysis, biotinylated proteins were collected by streptavidin-agarose beads. Western blotting of the Strep-pull-downs with the pT788/pT789 integrin β1 antibody identified a ~ 120–130 kDa protein with increased phosphorylation in PPM1F KO cells (Additional File 4: Fig. S4 C). Stripping and reprobing with the rabbit monoclonal integrin β1 antibody (clone D2E5) showed that both Strep-pull-downs contained equivalent amounts of integrin β1, which co-migrated with the biotinylated, pT788/pT789 integrin β1-reactive band (Additional File 4: Fig. S4 C). Integrin β1 immunoprecipitates contained the ~ 120–130 kDa form as the major protein, and this form of the protein was not modified by EndoglycosidaseH (EndoH)-treatment (Additional File 4: Fig. S4E). Together, these data suggest that either a fraction of mature integrin β1 or an integrin β1-associated protein with access to the surface of A172 cells and with an apparent molecular weight resembling integrin β1 is detected by the phospho-specific antibody. Apparently, the phosphorylation level of this protein is elevated in PPM1F-deficient cells indicating that this protein is either a direct PPM1F substrate or its phosphorylation is indirectly controlled by PPM1F.

With regard to functional consequences of *PPM1F* gene disruption, PPM1F KO cells exhibited increased adhesion to fibronectin, which was not compromised upon addition of the integrin αvβ3/αvβ5-specific inhibitor cilengitide (Fig. 7 B and C). In contrast, adhesion to vitronectin (VN), which could be completely abrogated by cilengitide, was unaltered in PPM1F KO cells (Fig. 7 B and C). These results demonstrate that A172 cell adhesion to fibronectin is strongly dependent on integrin β1 and integrin β1-mediated cell adhesion is under the control of PPM1F, while PPM1F deletion does not affect integrin αvβ5-mediated adhesion to vitronectin in this cell line, which expresses negligible levels of αvβ3 [26] (Fig. 7 B and C). To rigorously demonstrate that the lack of PPM1F is the primary cause of the PPM1F KO phenotype, we complemented the PPM1F KO1 cell line with human wildtype PPM1F fused to mKate2 (PPM1F KO + PPM1F-mKate2), the phosphatase inactive PPM1F D360A-mKate2 fusion protein (PPM1F KO + PPM1FDA-mKate2), or the fluorescent protein mKate2 only (PPM1F KO + mKate2) (Additional File 5: Fig. 5 A). Importantly, the lack of PPM1F resulted in a pronounced decrease in cell invasion (~ 50%), and this low-invasive phenotype was completely reverted by re-expression of wildtype PPM1F-mKate2, but not by the inactive PPM1F D360A (Fig. 7 D). Together, our data demonstrate that PPM1F expression levels directly impact the invasive potential of cancer cells, which might be due to the role of this protein phosphatase in integrin activity regulation.

### Increased integrin-based cell adhesion in PPM1F-deficient cells prohibits cell spreading despite elevated PAK activity

**Fig. 8:**
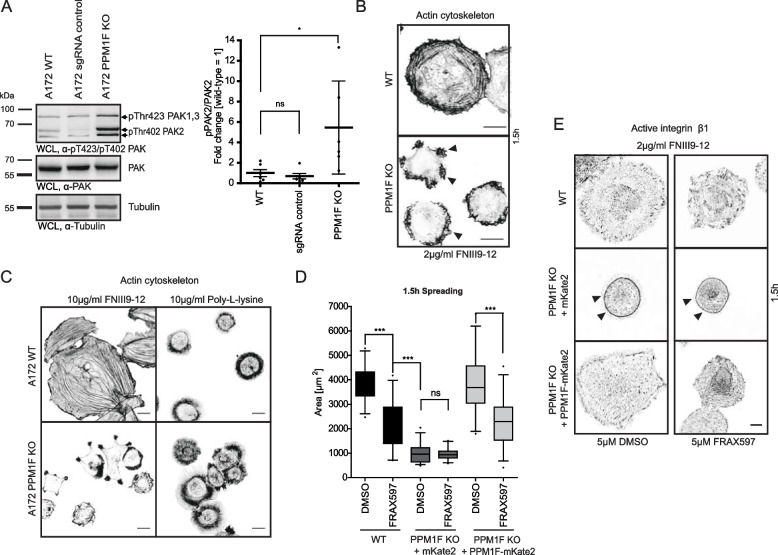
Increased integrin-based cell adhesion in PPM1F-deficient cells prohibits cell spreading despite elevated PAK activity. **A** Serum-starved A172 wildtype, sgRNA control and PPM1F KO cells [26] were seeded onto 2 µg/ml FN_III9-12_ for 45 min and WCLs were subjected to Western blotting with indicated antibodies (left panel). Graphs (right panel) show densitometric quantification of band intensities from pThr402PAK2 versus PAK antibody signal for the indicated samples from 5 independent experiments; wildtype was set to 1. Statistics were performed using one-way ANOVA, followed by Bonferroni post-hoc test (*p < 0.05, ns = not significant). **B** Serum-starved A172 wildtype and PPM1F KO cells were seeded onto 2 µg/ml FN_III9-12_ for 1.5 h, fixed and F-actin was stained. Samples were imaged using confocal microscopy. Representative pictures are shown; scale bar: 20 µm. **C** Cells as in (**B**) were seeded for 2 h on surfaces coated with 10 µg/ml fibronectin or poly-L-lysine, before fixation, F-actin staining and analysis by confocal microscopy; scale bar: 10 µm. **D** Spreading assays were performed with serum-starved A172 wildtype and PPM1F KO cells re-expressing mKate2 or re-expressing PPM1F-mKate2 cells, pre-treated with 5 µM DMSO or FRAX597 (PAK1-3 inhibitor) for 45 min in suspension before seeding onto 2 µg/ml FN_III9-12_ for 1.5 h. Cells were fixed, stained for F-actin and the covered area was quantified in ImageJ. Boxes and whiskers indicate median with 95% confidence intervals from two independent experiments; *n* ≥ 30 cells; dots indicate outliers. Statistics was performed using one-way ANOVA, followed by post-hoc Bonferroni test, (***p < 0.001, ns = not significant). **E** Serum-starved cells as in (**D**) were pre-treated with 5 µM DMSO or FRAX597 (PAK1-3 inhibitor) for 45 min in suspension before seeded onto 2 µg/ml FN_III9-12_ for 1.5 h. Cells were fixed and stained for active integrin β1. Cells were imaged by confocal microscopy. Representative pictures are shown; scale bar: 10 µm. See also Additional_File6 and Additional_File7

Besides integrin β1, PPM1F has additional cellular substrates. In particular the serine/threonine kinase PAK, which promotes cell motility by inducing membrane ruffles and cell protrusive activity, is dephosphorylated and inactivated by PPM1F [28, 37, 58, 59]. Accordingly, we investigated the activity status of PAK in PPM1F KO A172 cells. In line with prior studies, we observed a dramatic increase in phospho-PAK levels, in particular of PAK2 (~ eightfold), in PPM1F KO cells compared to wildtype or control cells indicating that loss of PPM1F results in constitutive PAK activity (Fig. 8 A). Upon plating of PPM1F KO cells, this elevated PAK activity was accompanied by disappearance of actin stress fibers and strong peripheral membrane ruffling, which was not observed in the wildtype cells (Fig. 8 B). While these effects on the actin cytoskeleton are known consequences of constitutive PAK activity, these processes are usually connected to reduced cell contractility and enhanced cell spreading [58, 60, 61]. Nevertheless, cell spreading of PPM1F KO cells, despite elevated PAK activity, was severely retarded, when cells were placed on an integrin ligand such as fibronectin (Fig. 8 C). In contrast, cell spreading was comparable between A172 wildtype and A172 PPM1F KO cells, when cells were placed on a poly-L-lysine substrate (Fig. 8 C). Therefore, the compromised initial spreading of PPM1F-deficient cells appears as a result of increased integrin function and occurs despite the elevated PAK-driven protrusive activity. This is particularly evident in live cell movies, where despite the highly dynamic lamellipodia and ruffles in the periphery of PPM1F-deficient A172 cells, these cells are not able to match the spreading seen in the wildtype cells (see Additional File 6 and 7: Movies 1 and 2). To firmly establish that enhanced integrin-based cell adhesion dominates PAK-driven cell protrusion, we measured cell spreading of wildtype, PPM1F KO and PPM1F KO + PPM1F-mKate2 cells in the presence and absence of a PAK inhibitor (FRAX5 97). As expected, in wildtype cells and in the PPM1F re-expressing PPM1F KO cells, inhibition of PAK resulted in reduced cell spreading consistent with the idea that PAK promotes cell protrusion (Fig. 8 D). In contrast, PPM1F KO cells, despite their constitutively elevated PAK activity, exhibited severely impaired cell spreading and this spreading defect was not altered by PAK inhibition (Fig. 8 D). As observed before [26], PPM1F deficiency resulted in a prominent circular accumulation of active integrin β1, and this “active integrin belt” appeared to limit further cell spreading (Fig. 8 E). Re-expression of PPM1F in the A172 PPM1F KO cells normalized the distribution of active integrin and the cells spread again comparable to wildtype A172 cells (Fig. 8 E). Most importantly, application of the PAK inhibitor partially reduced cell spreading in wildtype and PPM1F KO + PPM1F-mKate2 cells, while it did not alter the spreading defect of PPM1F KO cells and did not alter the prominent active integrin belt (Fig. 8 E). These findings help to segregate the effects of PPM1F deficiency on PAK or integrin β1. With respect to cell motility and invasive behaviour of glioblastoma cells, PPM1F reduces integrin activity to facilitate increased cell spreading and migration, thereby promoting the malignant behaviour of these cells.

### PPM1F determines A172 cell invasiveness in an in vivo CAM model

**Fig. 9:**
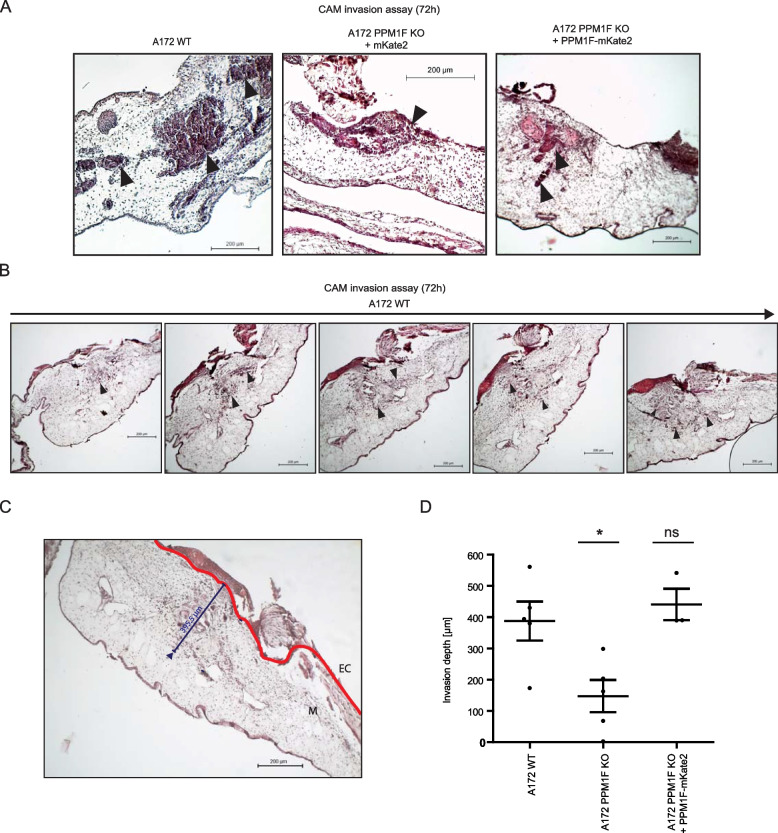
Presence of PPM1F determines the invasiveness of A172 cells in an in vivo CAM model. **A** Chicken chorioallantoic membrane (CAM) samples were inoculated on developmental day E9 with 1 × 10.^6^ A172 wildtype, A172 PPM1F KO, or A172 PPM1F KO cells re-expressing PPM1F-mKate2. Three days after tumor cell inoculation, the tissue was removed, fixed, paraffin-embedded, and 7 µm thick serial sections were made. Setions were H&E stained. Closed arrowheads point to tumor cells that have invaded the CAM mesoderm and locate in the vicinity of blood vessels, while the open arrowhead points to tumor cells remaining on top of the ectoderm; Shown are representative images; scale bar: 200 µm. **B** Representative serial sections from a CAM sample inoculated with A172 wildtype glioblastoma cells; arrowheads point to tissue-invaded cancer cells; scale bar: 200 µm. **C** Invasion depth was determined by measuring the orthogonal distance from the ectoderm (EC, red lines) reached by invasive cells in the mesoderm (M). For each series of serial sections, the highest invasion depth was determined; scale bar: 200 µm. **D** Invasion depth reached by the different cell lines was determined as in (**C**). Shown are individual values and means with 95% confidence intervals from *n* = 5 eggs per cell line. Statistics was performed using one-way ANOVA, followed by Bonferroni post-hoc test (*p < 0.05, ns = not significant). See also Additional_File5

To confirm the positive contribution of PPM1F to an invasive tumor phenotype in an intact tissue, we employed the chicken chorioallantoic membrane (CAM) assay, which monitors tissue infiltration by human tumor cells over the course of several days. To this end, we first compared the in vitro growth of A172 wildtype, PPM1F KO, or PPM1F KO + PPM1F-mKate2 cells (see Additional File 5: Fig. S5 C). While PPM1F KO cells were growing slightly slower than wildtype A172 cells, the re-constituted PPM1F KO + PPM1F-mKate2 cells were growing at a comparable rate as the PPM1F KO cells (see Additional File 5: Fig. S5 C). When placed onto the ectoderm of the chorioallantoic membrane (CAM) of fertilized chicken eggs, A172 wildtype cells invaded deep into the mesoderm over the course of three days (Fig. 9 A). In many instances, invasive groups of A172 cells were found adjacent to blood vessels (Fig. 9 A, arrowheads). Similarly, the PPM1F KO cells re-expressing PPM1F were found to penetrate the ectoderm and were reaching blood vessels, while A172 cells devoid of PPM1F stayed on top of the ectoderm, where they formed a single micro-tumor entity (Fig. 9 A, open arrowhead). To quantify the invasive potential of the cells, we generated serial sections of the CAM samples and determined the maximum distance of the invasive front, measured perpendicular from the ectoderm (Fig. 9 B and C). Importantly, while wildtype A172 cells and PPM1F KO cells re-expressing PPM1F WT were invading several 100 µm deep into the tissue, the PPM1F KO cells hardly penetrated the ectoderm and were not able to deeply infiltrate the mesoderm of the chicken CAM (Fig. 9 D). Together, our results demonstrate that PPM1F expression is directly linked to cancer cell invasiveness in an intact tissue and suggest that PPM1F-mediated control over integrin-dependent cell adhesion processes could promote the metastatic behaviour of tumor cells.

## Discussion

Protein phosphorylation and dephosphorylation are reciprocal post-translational modifications, which allow rapid and reversible adjustment of protein function by regulating enzyme activity or orchestrating protein–protein interactions. A conserved threonine motif in the integrin β1 cytoplasmic domain (T788/T789 in integrin β1) is known to affect integrin activity and this motif is regulated by phosphorylation in other integri β subunits [11]. We have found that the Mg^2+^/Mn^2+^-dependent protein phosphatase PPM1F targets these residues in vitro, thereby promoting filaminA-binding to the integrin β1 tail [26]. The abortion of embryonic development in PPM1F-deficient mice accompanied by misorganization of neuronal tissues and vascular defects now highlights PPM1F as an essential enzymatic regulator of integrin function in mammals. Together with the elevated adhesive phenotype of primary *ppm1f-/-* fibroblasts as well as PPM1F and filaminA knock-down neuro-epithelial cells these findings support the idea that integrin activity regulation by PPM1F is critical for efficient cell motility. Accordingly, the integrin-directed activity of PPM1F seems to promote the invasive potential of different cancer cells and unmasks a novel vulnerability of malignant tumors.

The pre-term death (E10.5) and the severe defects in the central nervous and cardiovascular system during embryogenesis of PPM1F-deficient mice demonstrate that this enzyme is critical for normal mammalian development and add further evidence to the idea that the negative regulation of integrin activity by PPM1F contributes to this essential function [26]. In this regard, it is interesting to contrast the severe consequences of PPM1F deficiency with the phenotypes of knock-out mice lacking other reported negative regulators of integrin activity such as ICAP-1, Sharpin, or Dok-1. For example, mice deficient for ICAP-1, an adaptor protein competing with talin for binding to the integrin β subunit, are viable, but show retarded growth and skeletal defects due to impaired osteoblast proliferation and differentiation [62, 63]. Mice lacking Sharpin, a further negative regulator of integrin activity and binding partner of the integrin α subunit [64], are viable and fertile, but sustain severe multi-organ inflammation and the development of secondary lymphatic organs is defective in these mice [65, 66]. Furthermore, Dok-1-/- mice do not have an obvious phenotype [67], but show elevated outside-in signaling through integrin αIIbβ3 in platelets [68]. As these integrin regulators are expressed in a wide variety of tissues, one must conclude that their integrin regulatory function is only needed in specific cell types or that their particular task is taken over by other proteins in situations such as tissue development and growth. In obvious contrast, PPM1F appears to perform non-redundant functions during embryonic development and, therefore, the *ppm1f-/-* phenotype has a high penetrance with malformations in multiple tissues. The drastic alteration seen in the central nervous system of *ppm1f-/-* embryos is of particular interest, as neuronal cells express PPM1E, a close homologue of PPM1F, which together share 66% sequence similarity in the central phosphatase domain [37, 69]. Our results now demonstrate that PPM1F function during mammalian development can not be compensated by other cellular serine/threonine phosphatases, not even by the related enzyme PPM1E.

In line with the idea that PPM1F-mediated dephosphorylation of the integrin β1 T788/T789 motif is a pre-requisite for filaminA association with the integrin cytoplasmic domain and filamin-mediated integrin inactivation [26] the phenotypes of PPM1F- and filaminA-knock-out mice show striking similarities. In particular, defective vascular development, hemorrhages and brain malformations as described here for PPM1F-/- embryos have also been reported for filaminA knock-out mice [43]. In addition, filaminA-deficient embryos show defects in neuronal progenitor cell emigration from the ventricular zone [70, 71], which was observed to a similar extent in embryos devoid of defined integrin α or β subunits [43, 72–74] and which is mirrored in PPM1F-/- embryos. Our findings of a gain-of-function with regard to integrin-mediated adhesion and a reduced haptotaxis migration in PPM1F- and filaminA-knock-down neuroepithelial cells now provide a mechanistic explanation for the accumulation of neuronal progenitor cells at the ventricular zone.

The phenotypic similarities between PPM1F-deficiency and filaminA-deficiency are not restricted to the murine system: Accumulation of neural progenitor cells in the ventricular region, as seen in PPM1F-deficient mouse embryos, is a hallmark of periventricular heterotopia (PH), a human condition associated with filaminA mutations [75]. While in male embryos loss-of-function mutations in the X-chromosomally encoded filaminA gene lead to lethality, females presenting with PH show neuronal cells remaining in nodular structures along the ventricle [75, 76]. The PH phenotype is thought to arise from random X-chromosomal inactivation of the remaining intact filaminA allele in heterozygous women. Accordingly, a portion of the neural progenitor cells behaves as functional filaminA knock-out cells with altered cell morphology and reduced radial migration [75, 76].

Similar to other protein phosphatases, PPM1F acts on multiple cellular substrates such as kinases, cytoskeletal proteins, and apoptosis regulators [71, 77]. Our prior in vitro results also indicate that the integrin β1 subunit is a further substrate of PPM1F. Nevertheless, it has to be noted that a phospho-threonine788/789 integrin β1 has not been observed in other studies [57]. While a phospho-threonine789 peptide of integrin β1 was reported in human liver samples by mass spectrometry [78], the regular detection of this phospho-modification is clearly hampered by shortcomings of existing reagents such as polyclonal phospho-specific antibodies. To address this problem, we have isolated integrin β1 from cells by immunoprecipitation and probed the integrin β1 precipitates with phospho-threonine-specific antibodies. Our results suggest that either threonine-phosphorylation of integrin β1 itself or threonine-phosphorylation of an integrin β1-associated protein with a similar apparent molecular weight is modulated by PPM1F. However, the reversible nature of protein phosphorylation, a presumably low stoichiometry of the phosphorylated integrin β1 subunit, and the limited capability of standard protein phosphatase inhibitors such as okadaic acid or calyculin A to interfere with PPM activity [79], render monitoring of integrin phosphorylation challenging. Therefore, it remains to be determined whether embryonic lethality of PPM1F knock-out mice is a consequence of alterations in the integrin phosphorylation status, integrin-filamin interaction, or integrin activity and to which extent deregulation of other PPM1F substrates may play a role. However, the phenotypic similarities upon disruption of genes encoding PPM1F, filaminA, and integrin subunits in mammals and the functional interplay of these proteins in intact cells strongly argue for a critical role of PPM1F-mediated integrin activity regulation in vivo.

Cell migration is not only critical for organ formation, efficient wound healing, or the immune response, but cell migration and tissue invasion are also prerequisites for cancer cell metastasis. In line with its role in integrin regulation, PPM1F is overexpressed in a number of highly motile and invasive human tumor types [28–35, 78]. Interestingly, the kinase PAK, which is a known regulator of the actin cytoskeleton and positively contributes to cell motility and invasion is also a substrate of PPM1F [37]. PPM1F-mediated dephosphorylation of conserved residues in the PAK kinase domain diminish PAK activity [79]. As constitutive PAK signaling has also been linked to cancer progression, it has been a conundrum, how the overexpression of PPM1F, a negative regulator of this kinase, could promote tumor metastasis [61]. Our findings now help to resolve this puzzle, as high PPM1F levels appear to support efficient cell motility by releasing integrin-based adhesive contacts. Conversely, a lack of PPM1F results in increased cell–matrix adhesion and suppression of cell spreading and motility despite increased PAK activity and despite numerous actin-based cell protrusions. The elevated levels of active integrin β1 found in PPM1F KO cells are not modulated by pharmacological inhibition of PAK, clearly segregating these two downstream targets of PPM1F. With regard to the motile behaviour of tumor cells, our results suggest that the integrin-directed activity of PPM1F has a dominant function and ultimately dictates the ability of transformed cells to overcome extracellular matrix barriers and to invade into tissue. This is underscored not only by in vitro matrigel invasion assays, but also by the aggressive infiltration of PPM1F-expressing glioblastoma cells into a regular 3-dimensional tissue in chicken embryos. Genetic ablation of PPM1F completely abrogates the ability of the glioblastoma cells to invade, while re-expression of PPM1F fully restores the metastatic phenotype without modulating the proliferation of the cells.

## Conclusions

Our results highlight a non-redundant role for the widely expressed serine-threonine phosphatase PPM1F in controlling integrin activity. As integrin activity regulation is central to many homeostatic processes in the human body, such as platelet aggregation, wound healing, or immune responses, further investigation of PPM1F and its interplay with substrates in adult tissues is warranted. Moreover, our findings provide a mechanistic explanation for the correlation between PPM1F expression and an invasive phenotype of tumor cells. Thus, the drugable enzyme PPM1F appears as an attractive novel therapeutic target to limit human tumor cell dissemination.

## Methods

### Antibodies

The following antibodies were used with the corresponding dilutions for western blot analysis (WB), immunofluorescence (IF), immunohistochemistry (IHC), immunoprecipitation (IP), or integrin activity assay (IA): α-Actinin (BM75.2, mouse anti-human, Abcam; 1:1000 WB), α_1_-integrin (TS2/7, mouse anti-human/anti-mouse, Abcam; 1:50 IF), α_2_-integrin (6 F1, mouse anti-human/anti-mouse, DSHB; 1:60 IF), α_3_-integrin (P1B5, mouse anti-human/anti-mouse, DSHB; 1:60 IF), α_5_-integrin (BIIG2, rat anti-human/anti-mouse, DSHB; 1:10 IF), α_v_-integrin (PE-P2 W7 mouse anti-human/anti-mouse, sc-9969; IF 1:300), β_1_-integrin (HMβ1-1, armenian hamster anti-mouse, Bio Legend; 1:300 IF; AIIB2, rat anti-human/anti-mouse, DSHB; 1:600 IF, IA; M-106, rabbit anti-mouse/anti-human, Santa Cruz; 1:500 WB; D2E5, rabbit anti-human, Cell Signaling; 1:1000 WB), human β_1_-integrin (P5D2, mouse anti-human, DSHB, 2.5 µg IP; 9EG7, rat anti- human, DSHB 2.5 µg IP; AIIB2, rat anti-human, DSHB; 2.5 µg IP), β_3_-integrin (2 C9.G3, arm. hamster anti-mouse, eBioscience; 1:300 IF; PM6/13, mouse anti-human, Abcam; 1:100 IF), β_5_-integrin (KN-52, mouse anti-mouse/human, eBioscience; IF 1:300), Focal adhesion kinase (FAK) (77, mouse anti-human, BD; 1:250 WB), integrin-linked kinase (ILK) (EP1593Y, rabbit anti-human, Epitomics; 1:800 WB), Kindlin-2 (3 A3, mouse anti-human, Millipore; 1:200 WB, 1:250 IF), Laminin (ab11575, rabbit anti-mouse, Abcam; 1:300 IHC), Nestin (rat-401, anti-mouse, Millipore; IHC 1:200), Paxillin (5H11, mouse monoclonal, Thermo Scientific; 1:1000 WB), hPPM1F (17,020–1-AP, rabbit anti-human, Protein-Tech; 1:1000 WB), mPPM1F (#1147, rabbit anti-mouse PPM1F; generated at the central animal care facility; University of Konstanz; 1:200 WB; see Additional File2: Fig. S2), FilaminA (EP2405Y, IgG, rabbit anti-human, Epitomics; 1:125.000 WB), Tubulin (E7, IgG1, mouse anti-human, DSHB; 1:1000), Talin (8 d4, mouse anti-human, Thermo Scientific; 1:800 WB, 1:40 IF), Vinculin (hVIN-1, mouse anti-human, Sigma; 1:2000 WB, 1:200 IF), Zyxin (Zol301, mouse anti-human, Abcam; 1:1000 WB), Dylight488-conjugated goat anti-mouse IgG (Jackson; 1:200), Cy3-conjugated goat anti-rabbit IgG (Jackson; 1:200), Cy3-conjugated goat anti-mouse IgG (Jackson; 1:200), Cy5-conjugated goat anti-mouse IgG (Jackson; 1:200), RhodamineRed-conjugated goat anti-rat IgG (Jackson; 1:200), RhodamineRed-conjugated goat anti-Armenian Hamster IgG (Jackson; 1:200), HRP-conjugated goat anti-mouse IgG (Jackson; WB 1:10 000), HRP-conjugated goat anti-rat IgG (Santa Cruz; 1:250), HRP-conjugated goat anti-rabbit IgG (Jackson; WB 1:3000), unspecific control IgG (anti-mouse, 96/1, generated at the Tierforschungsanlage; University of Konstanz; anti-rat, MJ7/18 Endoglin, DSHB).

### Husbandry and genotyping of mice

Mice were kept in accordance with relevant institutional and national guidelines and regulations in the central animal care facility of University of Konstanz. The B6.129P2-PPM1Ftm1Dgen/J (PPM1F ±) mouse strain was obtained from The Jackson Laboratory (Bar Harbor, ME). The targeted *ppm1f* gene was created by Deltagen (San Mateo, CA) by inserting a Lac0-SA-IRES-lacZ-Neo555G/Kan cassette via homologous recombination into the *ppm1f* locus allowing the endogenous promoter to drive expression of β-galactosidase. The PPM1F ± mice have been backcrossed at least 20 generations to C57BL/6 mice. 3 week old mice or embryos were genotyped by amplification of DNA extracted from tissue biopsies or isolated from mouse embryonic fibroblast. The following PCR primers were used:

Primer 1: wild type forward: 5’ –CAACTCTCCATCATGCCCATCAG– 3’.

Primer 2: common reverse: 5’ –AAGCAGGAAGGGACACGTGTCGGTC– 3’.

Primer 3: targeted allele forward: 5’- GGGTGGGATTAGATAAATGCCTGCTCT– 3’.

For genotyping, a PCR with 32 cycles was performed with an annealing temperature of 59 °C and an elongation time of 40 s at 72 °C yielding a 200 bps and 450 bps PCR fragment for the wildtype and the targeted allele, respectively (Fig. 1A) [26]. For timed matings, heterozygous *ppm1f* ± mice were allowed to mate overnight. 14.5., 13.5, 12.5, 11.5, or 10.5 days post coitus, the pregnant female mouse was anesthetized with isoflurane and directly sacrificed by cervical dislocation. For each time point more than 10 embryos were genotyped.

### LacZ staining of frozen tissue sections

*Ppm1f* + */* + and *ppm1f* ± mice were anesthesized and sacrificed as above and perfused with phosphate-buffered saline (PBS) for 2 min followed by 4% Paraformaldehyde (PFA) in PBS for 5 min. Tissue was dissected and placed into 4% PFA in PBS at 4 °C. The fixed tissue was washed with PBS and cryo-protected via a sequential transfer to 10% sucrose in PBS overnight at 4 °C, 20% sucrose for 3 h at 4 °C, and 30% sucrose for 3 h at 4 °C. Samples were embedded in O.C.T Tissue-Tek (Sakura Finetek, Staufen, Germany) using histology moulds and stored at −80 °C. 10 µm thick tissue sections were cut using a cryostat CM1900 (Leica, Wetzlar, Germany) at −25 °C and transferred to poly-L-lysine-coated slides. Dried sections were washed 3 × in PBS and 1 × in ddH_2_O for 5 min. The 5-bromo-4-chloro-3-indolyl-β-D-galactopyranoside (X-gal) dilution buffer (0.02% NP40, 2 mM MgCl_2_, 0.01% sodium deoxycholat, 5 mM potassium ferricyanide and 5 mM potassium ferrocyanide, pH 7.3) was pre-warmed up to 37 °C before the substrate X-gal (1 mg/ml in N,N-diemthylformamide) was added. After incubation overnight at 37 °C, sections were washed in PBS and in ddH_2_O each for 5 min, dried and mounted with mounting medium (Dako, Glostrup, Denmark).

### Whole-mount detection of β-galactosidase activity in mouse embryos

Heterozygous *ppm1f* ± mice were allowed to mate overnight. 10.5 days post coitus the female mouse was anesthesized and sacrificed as above. The embryos were dissected and fixed in β-galactosidase fixative (PBS pH 7.4, 0.2% glutaraldehyde, 1.5% formaldehyde, 5 mM EGTA and 2 mM MgCl_2_) for 90 min at room temperature (RT). Embryos were washed 3 × in β-galactosidase wash buffer (PBS pH 7.4, 2 mM MgCl_2_, 0.02% NP40 and 0.01% sodiumdeoxycholate) for 20 min at RT with soft end-to-end rocking. Afterwards, the embryos were incubated in β-galactosidase staining solution (β-galactosidase wash buffer supplemented with 1 mg/ml X-gal, 5 mM potassium ferricyanide and 5 mM potassium ferrocyanide) for 18 h at 30 °C in the dark. Next, embryos were washed 3 × in PBS for 20 min each and photographed. Stained embryos were post-fixed with 3% formaldehyde and 2% glutardialdehyde in PBS pH 7.4 for 2 h at 4 °C. Afterwards, the samples were rinsed for 4 h at RT, dehydrated and embedded in paraffin. 14 µm thick tissue sagittal sections were prepared using a microtome RM2125RT (Leica) and mounted on microscope slides (HistoBond +, Marienfeld, Lauda Königshofen, Germany). Slides were dewaxed four times for 5 min by Histoclear (National Diagnostics, Atlanta, GA) and afterwards embedded with Roti-Histokitt (Carl Roth, Karlsruhe, Germany).

### Immunofluorescence staining of frozen mouse embryonic tissue sections

Heterozygous *ppm1f* ± mice were allowed to mate overnight. 10.5 days post coitus the female mouse was anesthesized and sacrificed as above. The embryos were dissected and fixed in 4% PFA overnight at 4 °C. The embryos were washed with PBS and cryo-protected in 10% sucrose in PBS. The embryos were transferred to a 20% sucrose solution with subsequent incubation in 30% sucrose solution for about 2 h each at 4 °C. The samples were then incubated in 30% sucrose and O.C.T Tissue-Tek (1:1) for 1 h at RT with soft end-to-end rocking. Finally, the embryos were embedded in O.C.T Tissue-Tek using histology moulds, transferred to dry ice and stored at −80 °C.

Sections were cut using a cryostat (temperature of chamber: −30 °C; temperature of object: −25 °C) at 12 µm thickness and transferred to adhesion microscope slides (HistoBond +, Marienfeld, Lauda Königshofen, Germany). Sections were dried at 30 °C for 2 h and transferred to −20 °C for storage.

At the day of use, sections were allowed to thaw at RT for several minutes, washed in PBS, blocked (PBS + 1% bovine serum albumin (BSA) + 1% heat inactivate calf serum (CS) + 0.1% TritonX100) for 30 min followed by incubation with first antibody in blocking solution overnight at 4 °C. Samples were washed, incubated with the corresponding second antibody (all 1:200) for 1 h at RT and washed again. After incubation in DAPI solution (1:2000) for 20 min and a last washing step, sections were mounted with Dako mounting medium.

### Nissl staining of brain sections

Ppm1f +/+ and ppm1f ± mice were anesthesized and sacrificed as above and perfused with PBS for 2 min, then for 5 min with 3% formaldehyd and 2% glutardialdehyd in PBS. Brains were excised and placed into 3% formaldehyde and 2% glutardialdehyde in PBS, pH 7.4 overnight at 4 °C. Fixed brains were rinsed with PBS, dehydrated and embedded in paraffin. 20 µm thick coronal sections were prepared using a microtome RM2125RT (Leica). The sections were transferred to coated slides (HistoBond +, Marienfeld, Lauda Königshofen, Germany), de-waxed by four passages through Histoclear (National Diagnostics, Atlanta, GA) for 5 min and hydrated in MilliQ water for 30 min. Slides were stained with 0.1% cresyl violet acetate (Sigma, St. Louis, MO) for 4 min under agitation. Slides were dehydrated for 2 min using 70% EtOH (twice), 95% EtOH (twice), 100% EtOH (twice), cleared in xylene for another 2 min, and finally mounted with Roti-Histokitt (Carl Roth, Karlsruhe, Germany).

### Human PPM1F tissue expression data analysis

Human PPM1F RNA-sequencing tissue data (reported as mean protein-coding transcripts per million (pTPM)) were purchased from the Human Protein Atlas portal (HPA transcriptomic study) (https://www.proteinatlas.org/ENSG00000100034-PPM1F) [80, 81], the Genotype-Tissue Expression project (GTEX; https://www.gtexportal.org/home/gene/ENSG00000100034), the Functional Annotation of Mammalian Genome 5 (FANTOM5) project (https://fantom.gsc.riken.jp/5/) [82, 83] and the BioGPS Gene Atlas (http://biogps.org/#goto=genereport&id=9647, Dataset U133 A, gcrma) [83]. Data of analyzed tissues were referenced to the highest PPM1F messenger RNA (mRNA) level measured in the corresponding study, which was set to 100%. Only data from tissues apparent in at least three of the four studies were combined in the final figure and sorted from the highest to the lowest expression levels occurring in one of the four studies.

### Procurement of primary human and murine cells

Primary human blood cells (PBMCs and granulocytes) were isolated from healthy human donors essentially as described [84]. Primary normal human dermal fibroblasts isolated from juvenile foreskin were obtained from Promocell (Promocell, Heidelberg, Germany), human primary hepatocytes were provided by R. Lambrecht [85], human brain microvascular endothelial cells were provided by A. Schubert-Unkmair [86], human vaginal epithelial cells (MS74) were obtained from A.J. Schaeffer (Feinberg School of Medicine, Northwestern University, Chicago, IL), human urethral cells were purchased from Cell Applications (San Diego, CA) and their lysates were provided by R. Lambrecht (University of Konstanz, Germany).

Isolation of murine skin endothelial cells was performed as described by Henrot et al. [87]. To this end, mice were sacrificed via cervical dislocation, the tail of the mice was cut and the skin was peeled off. The isolated skin was soaked in 70% ethanol and rinsed in cold Hepes-buffered salt solution, cut in 3 pieces of similar size and incubated in 4 mg/ml dispase in DMEM supplemented with 10% FCS and 1% non-essentail amino acids (NEAA) for 90 min at 37 °C and 5% CO_2_. The epidermis was separated from the dermis using forceps and the dermis was transferred into a dish containing Q-medium (DMEM, 10% FCS, 1% NEAA, 1% Pen/Strep, 20 µg/ml ciprofloxacin) with the basal lamina side oriented upwards. Endothelial cells were extracted by scratching the dermal tissue with a closed pair of forceps. The supernatant was collected and filtered through a 70 µm cell strainer. After centrifugation, the cell pellet was suspended into Q-medium supplemented with 1% insulin. Streptavidin-Dynabeads (Invitrogen) coupled to biotinylated rat anti-mouse CD31 antibody (BD Pharmingen) were incubated for 1 h at 4 °C with the cell suspension [88], washed, and bead-associated cells were seeded onto 0.1% gelatine.

### Cell Culture and transient transfection

Human embryonic kidney 293 T cells (ATCC CRL-3216), SK-N-MC neuroepithelial cells (ATCC HTB-10), SH-SY5Y neuroblastoma cells (DSMZ ACC 209); MDA-MB-231 breast adenocarcinoma (ATCC CRM-HTB-26), MCF-7 breast adenocarcinoma (ATCC HTB-22), HEPG2 hepatoma cells (ATCC HB-8065), A549 lung carcinoma (ATCC CCL-185), DLD-1 colorectal adenocarcinoma (ATCC CCL-221), HCT 116 colorectal carcinoma (ATCC CCL-247), HT-29 colon adenocarcinoma (ATCC HTB-38), SW480 colon adenocarcinoma (ATCC CCL-228), U-138 MG glioblastoma (ATCC HTB-16), A172 glioblastoma cells (ATCC CRL-1620) and genetically modified A172 cells [26] were grown in DMEM supplemented with 10% fetal calf serum.

AGS gastric adenocarcinoma (ATCC CRL-1739), Jurkat T cell leukemia (DSMZ ACC 282), and THP-1 acute monocytic leukemia cells (DSMZ TIB-202) were grown in RPMI 1640 supplemented with 10% fetal calf serum.

Mouse embryonic fibroblasts (MEFs) were isolated from timed matings of heterozygous mice at day 10.5 day post coitus and immortalized via transduction with pBabeZeo SV40 largeT (Addgene, plasmid #1779) [26]. MEFs and NIH3 T3 cells were cultured in DMEM supplemented with 10% fetal calf serum, non-essential amino acids and sodium pyruvate. All cells were maintained at 37 °C, 5% CO_2_ and sub-cultured every 2–3 days.

For transient transfection of 293 T cells, cells were seeded at 25% confluence the day before in 10 cm petri dishes and transfected using standard calcium phosphate method with a total amount of 5 µg plasmid DNA/dish.

### Whole cell lysates and Western blotting

To obtain whole cell lysates (WCL), equal cell numbers were lysed by treatment with RIPA-buffer (1% Triton X-100, 50 mM HEPES, 150 mM NaCl, 10% glycerol, 1.5 mM MgCl_2_, 1 mM EGTA, 0.1% w/v SDS, 1% v/v Deoxycholic acid) supplemented with freshly added protease and phosphatase inhibitors (10 mM sodium pyrophosphate, 100 mM NaF, 1 mM sodium orthovanadate, 5 μg/ml leupeptin, 10 μg/ml aprotinin, 10 μg/ml pefablock, 5 μg/ml pepstatin, 10 μM benzamidine) and phosphatase saturating substrate (para-nitrophenolphosphate [pNPP], Sigma-Aldrich, 10 mM). Chromosomal DNA and cell debris were pelleted by addition of sepharose beads and centrifugation (13,000 rpm, 30 min, 4 °C). Supernatant was supplemented with 2 × or 4 × SDS sample buffer (2 or 4% wt/vol SDS, 20% wt/vol glycerol, 125 mM Tris–HCl, 10/20% vol/vol β-mercaptoethanol, 1% wt/vol Bromophenol blue, pH 6.8) and boiled for 5 min at 95 °C. The protein amount was adjusted via the bicinchoninic acid protein assay kit (Pierce; Thermo Fisher Scientific) according to the manufacturer’s protocol and analysed by Western blot using pre-stained marker as protein size control (26,619 Thermo Fisher Scientific). Briefly, proteins were resolved on 8–18% sodium dodecyl sulfate polyacrylamide gel electrophoresis (SDS-PAGE =. After separation, the proteins were transferred to a polyvinylidene fluoride membrane (Merck Millipore), followed by blocking in 2% BSA containing 50 mM Tris–HCl, 150 mM NaCl, and 0.05% Tween 20, pH 7.5 (TBS-T) buffer. The membrane was incubated with primary antibody in blocking buffer overnight at 4 °C, washed three times with TBS-T, and incubated with HRP-conjugated secondary antibody in TBS-T for 1 h at RT. The chemiluminescent signal of each blot was detected with ECL substrate (Thermo Fisher Scientific) on the Chemidoc Touch Imaging System (Bio- Rad) in signal accumulation mode. Acquired images were processed in Adobe Photoshop CS4 by adjusting illumination levels of the whole image.

### Lentiviral production and generation of stable cell lines

Lentiviral particles were produced as described previously [89]. Briefly, 293 T cells were transfected by standard calcium-phosphate co-precipitation using 3.5 µg pMD2.G (packaging cassette), 5 µg psPAX2 (viral envelope expression cassette) and 6.5 µg pLKO.1, pLenti CRISPRv2, or pUltra containing the desired small hairpin RNA (shRNA), single guide RNA (sgRNA), or cDNA, respectively. After 72 h, virus containing culture supernatant was collected, ultra-centrifuged and target cells were infected with virus concentrate by spinfection (1 h, 800 g, RT) with 8 µg/ml polybrene following incubation for 24 h at 37 °C. Control cells were generated by transducing cells with virus harboring scrambled shRNA pLKO.1 or empty pUltra vector. After 48 h recovery time, SK-N-MC cells transduced with pLKO.1 and SH-SY5Y cells transduced with pLenti CRISPRv2 sgRNA-PPM1F were selected with puromycin (1 μg/ml, 6 days) to result in mixed populations of stable knock-down or knock-out cells, which were used in experiments. MEF cells transduced with pUltra-derived vectors were selected for fluorescence protein expression by flow cytometry and mixed populations of GFP-expressing wildtype (MEF PPM1F +/+), PPM1F KO (MEF PPM1F-/-), PPM1F KO re-expressing wildtype human PPM1F (MEF PPM1F-/- hWT), or PPM1F KO re-expressing inactive human PPM1F D360A (MEF PPM1F-/- hDA) were employed in experiments. The generation of A172 Cerulean expressing, control (sgCerulean), PPM1F KO (sgCerulean, sgPPM1F) and PPM1F KO cells re-expressing mKate2, mKate2-PPM1F or mKate2-PPM1FD360 A was described previously [26]. Single cell clones of stably transduced A172 cells were grown up in regular growth medium supplemented with 20% FCS, penicillin/streptomycin, and 20% conditioned medium, expanded and used in experiments.

### Generation of knock-down SK-N-MC cells and knock-out SH-SY5Y cells

For the generation of recombinant, shRNA-expressing lentiviral particles, the shRNA vector system pLKO.1 developed by Stewart and colleagues [90] was applied as described previously for HEK293 T and A172 cells [26]. The different shRNAs were designed by using the AAN19 algorithm and small interfering RNA (siRNA) selection program of the Whitehead Institute for Biomedical Research (http://sirna.wi.mit.edu/). According to the prediction of the siRNA selection program two complementary oligos were synthesized, respectively.

hFilaminA_shRNA_sense.

5’-ccggaaGACCACCTACTTTGAGATCctcgagGATCTCAAAGTAGGTGGTCtttttttg-3’.

hFilaminA_shRNA_anti.

5’-aattcaaaaaaaGACCACCTACTTTGAGATCctcgagGATCTCAAAGTAGGTGGTCtt-3’.

hPPM1F_shRNA_sense.

5’-ccggaaCCAGCTCTTCGGCTTGTCTctcgagAGACAAGCCGAAGAGCTGGtttttttg-3’.

hPPM1F_shRNA_anti.

5’-aattcaaaaaaaCCAGCTCTTCGGCTTGTCTctcgagAGACAAGCCGAAGAGCTGGtt-3’.

The oligos were annealed and cloned via AgeI and EcoRI restriction sites into plasmid pLKO.1 puro (Addgene; plasmid #8453), which provides puromycin resistance for selection of stable knock-down cells. The correct insertion of the shRNA cassettes was verified by sequencing.

For the generation of recombinant, Cas9 and sgRNA-expressing lentiviral particles, the CRISPR/Cas9 vector system pLenti CRISPR v2 was used (a kind gift from Feng Zhang; Addgene #52,961) [91]. To this end, plentiCRISPRv2 was digested with BsmBI and then ligated with a double-stranded sgRNA oligopair targeting PPM1F [26] to generate pLenti CRISPRv2 sgRNA-PPM1F. The correct insertion of the sgRNA was verified by sequencing.

### Complementation of *ppm1f-/-* MEF cells

For the re-expression of PPM1F in *ppm1f-/-* mouse embryonic fibroblasts the human PPM1F or human PPM1F D360 A cDNA [26] was amplified via the following primers: PPM1F-XbaI-sense: 5’-TGCATCTAGAATGTCCTCTGGAGCCCCAC–3’ and PPM1F-BamHI-anti: 5’-CAGTAAGGATCCCTAGCTTCTTGGTGGAG–3’. The resulting PCR fragments were cloned into pUltra (a gift from Malcolm Moore, Addgene plasmid #24,129) via XbaI and BamHI restriction sites leading to a bi-cistronic expression of the human PPM1F wildtype and green fluorescent protein (GFP) or human PPM1F D360 A and GFP. The empty vector was used to generate *ppm1f* + */* + and *ppm1f-/-* control cell lines expressing only GFP. Plasmid constructs were verified by sequencing.

### Immunostaining for confocal microscopy and cell spreading analysis

Sterile coverslips were coated with PBS containing 0.4–10 μg/ml Glutathion-S-transferase (GST)-tagged fibronectin type III repeats 9–11 (FN_III9-11_; gift of M.A. Schwartz, Yale University, NewHaven, CT) or 10 µg/ml poly-L lysine (SERVA, Heidelberg, Germany) as integrin-independent control overnight at 4 °C in a 24-well plate and cells were starved with DMEM + 0.5% FCS for 15 h. The next day, coating solution was removed and wells were blocked with suspension medium (DMEM + 0.25% BSA). In parallel, cells were trypsinized, trypsin-inactivated with soybean trypsin inhibitor (AppliChem, Darmstadt, Germany, 12.5 mg in 50 ml DMEM, sterile filtered), counted and kept in suspension medium for 45 min at 37 °C. In experiments using the PAK1-3 inhibitor FRAX597 (CAS 1286739–19-2, Selleckchem Houston, USA), cells were pre-treated with 5 µM inhibitor or DMSO as control in suspension for 1 h at 37 °C, before 2.5 × 10^4^ cells were seeded onto coverslips and allowed to adhere for corresponding time periods. Cells were fixed with 4% PFA supplemented with 0.1% Triton X-100 for 5 min at RT and again without Triton for 20 min. Coverslips were washed thrice with PBS^++^ (0.9 mM CaCl_2_, 0.5 mM MgCl_2_ in 1 × PBS) and blocked for 20 min with blocking solution (10% CS in PBS). Primary antibody solution was added for 1 h at RT. After washing thrice with PBS^++^ and blocking for another 20 min, coverslips were treated with secondary antibody in blocking solution and optionally with phalloidin-Cy5 or DAPI for 1 h at RT in the dark. Finally, cells were washed thrice with PBS^++^ and mounted with Dako fluorescent mounting medium (Dako, Glostrup, DK). Samples were imaged on a LEICA SP5 confocal microscope equipped with a 63.0 ×/1.40 NA oil HCX PL APO CS UV objective and acquired in xyz mode with 1024 × 1024 pixel format and 100 Hz scanning speed at 8 bit resolution. All images were analyzed in ImageJ Software. For spreading assays, a macro was set-up together with the Bioimaging Center (BIC) at the University of Konstanz and used for quantitative picture analysis. Unrecognized cells were analyzed manually in the Leica LAS AF Lite software.

### Immunostaining for FACS analysis

Cells were detached with trypsin/EDTA, pelleted and suspended in FACS buffer (0.1% NaAzide, 5% FCS in PBS). After centrifugation (800 rpm, 3 min) cells were washed with FACS buffer and 3 × 10^5^ cells were transferred into Eppendorf tubes respectively, centrifuged (2500 rpm, 2 min, 4 °C) and incubated with primary antibody in FACS buffer at desired concentration for 1 h at 4 °C under constant rotation. Cells were washed thrice with FACS buffer and incubated with secondary antibody in FACS buffer at desired concentrations for 30 min at 4 °C under constant rotation in the dark. After washing thrice with FACS buffer cells were suspended in 1 ml of FACS buffer containing 2 mM EDTA. Finally, cells were analyzed by flow cytometry (BD LSRII, FACSDiva™ software, BD Biosciences, Heidelberg, Germany).

### Replating assay for pT788/pT789 β1 integrin and pT423 PAK1, 3/pT402 PAK2 analysis in intact cells

10 cm dishes were coated with 2 μg/ml FN_III9-11_ in PBS overnight at 4 °C. Cells were starved with DMEM + 0.5% FCS overnight at 37 °C. The next day, dishes were blocked with DMEM + 0.25% BSA for 1 h at 37 °C. In parallel, cells were trypsinized, trypsin-inactivated with Soybean inhibitor (AppliChem, 12.5 mg in 50 ml DMEM, sterile filtered), counted and kept in suspension medium for 45 min at 37 °C. Afterwards, the same cell numbers were seeded onto coated dishes and allowed to adhere for corresponding time periods at 37 °C. Dishes were washed with PBS, whole cell lysates prepared and subjected to Western blot analysis. Phosphorylation of proteins was detected by phospho-specific antibodies. Densitometric analysis was performed by ImageJ software. The amount of phosphorylated β1 integrin or PAK was normalized to total β1 integrin or PAK expression levels.

### Cell adhesion assay

96-well plates were coated with PBS containing corresponding concentrations of FN_III9-11_ or poly-L lysine (SERVA, Heidelberg, Germany) as integrin-independent control overnight at 4 °C. Wells were blocked with DMEM + 0.25% BSA for 1 h at 37 °C. In parallel, serum-starved cells were trypsinized and kept in suspension medium for 45 min. 2.5 × 10^4^ cells/well were seeded and allowed to adhere for the indicated time periods at 37 °C. After incubation, non-adherent cells were removed by gently washing with PBS^++^ thrice. Adherent cells were fixed with 4% PFA in PBS for 15 min, washed with PBS and stained with 0.1% crystal violet in 0.2 M borate buffer (pH = 8.5) for 30 min. After intense washing, the color was unhinged from cells with 10 mM acetic acid and the absorption was measured at 590 nm using a spectrophotometer.

### Integrin activity assay

Serum starved cells were trypsinized and kept in suspension (2% BSA, 5 mM glucose in PBS) for 45 min before they were stimulated with 10 µg/ml FN_III9-11_ for 15 min at 37 °C or kept unstimulated by adding ddH_2_O. Cells were put on ice and split into two fractions, which were either stained for active integrin β1 (9EG7 1:600) or total integrin β1 (HMβ1-1 1:300 or AIIB2 1:600) for 1 h on ice in PBS + 2% BSA. Cells were washed thrice with PBS and incubated with Rhodamine-Red conjugated secondary antibody for 45 min on ice in the dark. Cells were washed and fluorescence intensity was measured by flow cytometry (BD LSRII, FACSDiva™ software, BD Biosciences, Heidelberg, Germany).

### Surface biotinylation, integrin β1 immunoprecipitation, and EndoH treatment

A172 cells were starved overnight in DMEM, 0.5% FCS. 1 × 10^6^ starved cells were seeded onto fibronectin-coated plates and incubated for 45 min. Medium was removed and cells were either biotin labeled before lysis using 2 µg/ml EZ-Link Sulfo-NHS-LC-Biotin (ThermoFisher) in PBS for 15 min at 4 °C or cells were directly lysed in RIPA lysis buffer supplemented with protease and phosphatase inhibitors including 10 mM of the phosphatase saturating substrate para-nitrophenolphosphate (pNPP, Sigma-Aldrich) as described above. For immunoprecipitation, the RIPA lysis buffer was further supplemented with 10 µM of compound 16 (MolPort-008–312–685, MolPort EU, Riga, Latvia), a broad-spectrum phosphatase inhibitor [92]. Lysates were sonicated (10 pulses at 50%), then centrifuged at 13,000 rpm for 30 min at 4 °C. For immunoprecipitation, 3 µg of the indicated monoclonal antibodies were incubated for 3 h at 4 °C while rotating, before 10 µl of Protein A/G beads (Santa Cruz Biotechnology, Santa Cruz, CA) were added for 1 h. For EndoH treatment of immunoprecipitated integrin β1, beads were washed twice with RIPA buffer and then washed once with 1 × PBS, incubated with Glycoprotein Denaturing Buffer, before incubation with EndoH (New England Biolabs, Ipswich, MA) at 37 °C for 1 h according to manufacturers recommendations. Biotinylated proteins were collected by adding streptavidin beads to the whole cell lysates for 1 h at 4 °C while rotating. Beads were washed twice using RIPA lysis buffer, before the pellet was boiled at 95 °C for 5 min in 2 × SDS loading buffer.

### Single cell tracking

MEFs were seeded in 24 well plates and incubated for 24 h. Cells were starved in DMEM supplemented with 0.5% BSA for 12 h, afterwards stimulated with growth medium and imaged for 12 h (30 min/frame). Single cells were tracked manually using ImageJ particle tracking plugin and analyzed using the chemotaxis and migration tool (Ibidi GmbH, München, Germany).

### Holotomographic live-cell imaging

35 mm µ-dishes (ibidi, Gräfelfing, Germany) were coated with 2 µg/ml FN_III9-11_ in PBS overnight at 4 °C. Cells were serum starved (DMEM + 0.5% FCS) for max 12 h. After the coating solution was removed, dishes were blocked with suspension medium (DMEM plus 0.25% BSA) and prewarmed to 37 °C for 1 h. In parallel, cells were trypsinized, cells were suspended in DMEM + soybean trypsin inhibitor (12.5 mg in 50 ml DMEM, sterile filtered; AppliChem), counted and kept in suspension medium for 45 min at 37 °C. 1 × 10^5^ cells were seeded in µ-dishes in a total volume of 1.5 ml suspension medium. Image acquisition started 30 min after cell seeding under controlled temperature and atmosphere conditions at 5% CO2 and 37 °C using a live cell 3D holotomographic microscopy (CX-A, Nanolive, Tolochenaz, Switzerland). Images were taken every 4 min. Brightness and contrast was adjusted equally over the whole image and images were sharpened using ImageJ.

### Haptotaxis migration and 3D matrigel invasion assays

Haptotaxis and invasion assays were performed similar as described previously [93, 94]. Shortly, for haptotaxis assays the lower side of the membrane of modified Boyden chambers (Millicell, 8-µm pore size, 12-mm diameter; Millipore, Bedford, MA) was coated with 10 µg/ml or 40 µg/ml of fibronectin, or 2% BSA as indicated for 1 h at RT. The chambers were then placed in a 24-well plate and 0.4 ml of medium with 0.25% BSA was added to the lower compartment. For invasion assays, growth-factor reduced Matrigel (CAS 356231, Corning, NY, USA) was diluted in serum-free DMEM on ice to yield 30 µg Matrigel in 100 µl total volume and was added to the topside of modified Boyden chambers (Millicell, 8-µm pore size, 12-mm diameter; Millipore, Bedford, MA), which were placed in a 24-well plate. The matrigel was allowed to polymerize for 30 min at 37 °C, before 0.4 ml of migration medium with BSA as control or 20% FCS was added to the lower compartment.

In both assays, serum-starved cells (0.5% FCS, 18 h) were added to the upper compartment (8 × 10^4^ cells in 0.3 ml medium w/o FCS) and after 6 h (haptotaxis assays) or 24 h (invasion assays) at 37 °C, chambers were washed with PBS and cells fixed by treatment with 4% PFA in PBS. Cells on the upper membrane surface were removed by a cotton tip applicator, cells were stained with 0.1% crystal violet in 0.2 M borate buffer (pH = 8.5) for 30 min, and migration values were determined by dye elution with 10% acetic acid and absorbance measurement at 590 nm or by counting five fields/chamber under the light microscope. Mean values were obtained from three individual chambers per sample condition per assay.

### In vitro wound healing assay

1 × 10^6^ SH-SY5Y cells were seeded into 6-well plates and incubated for 2 days until they grew confluent. Cell were starved in DMEM, 2% FCS (starvation medium) for 16 h before the confluent cell monolayer was scratched by using a pipette tip. Cells were washed once and starvation medium was added. Images were taken with the 4 × objective of the Nikon ECLIPSE TS100 microscope equipped with NIS-Elements F 4.30.01 software. Scratches were imaged at 5 positions, the cell-free area was traced using ImageJ and the distance between wound margins was determined.

### Egg preparation and chorioallantoic membrane (CAM) assay

Fertilized chicken eggs were purchased from LSL-Rhein-Main Geflügelvermehrungsbetriebe GmbH, cleaned with warm water and incubated for 8 days after breeding at 37 °C with 50–70% humidity. Similar to previous studies [95–97], eggs were opened at embryonic development day 8 (EDD8) between the two main blood vessels to create a 1 cm^2^ big window for tumor cell inoculation, after the air sac was re-located by vacuum application. Eggs were covered with tape and incubated until the next day (EDD9). A total of 1 × 10^6^ serum-starved A172 wildtype or PPM1F KO cells were re-suspended in 20 µl PBS, mixed with 20 µl growth factor-free Matrigel (CAS 356231, Corning, NY, USA) and added on top of the CAM into a silicon ring, before covering the window again with tape. After an incubation of 3 days, chicken embryos were numbed with anaesthetic solution (100 mg/ml benzocaine in DMSO). The CAM was removed and fixed overnight in fixation solution (2% PFA, 2% glutardialdehyde, 0.1 M NaPO_4_ buffer, pH 7.3). Samples were paraffine-embedded, 7 µm thick sections were made and stained with H&E staining before being imaged at the Bioimaging Center (BIC, University of Konstanz, Germany) at a Leica DME microscope equipped with Leica LAS EZ software.

### Statistics

All data are presented as mean ± standard error of the mean (SEM) or mean ± standard deviation (SD) as indicated. All statistical significances were determined using a two-tailed Student's t-test or one-way ANOVA followed by Bonferroni post-hoc test with Prism5 (GraphPad, La Jolla, CA, USA). Significance is indicated with * = *p* < 0.05, ** = *p* < 0.01, *** = *p* < 0.001 or ns = not significant.

## Supplementary Information

Additional file 1: Figure S1 indicates similar size and brain histology of PPM1F ± mice compared to their wildtype littermates. Figure S1: Related to Fig. 3. Ppm1f ± adult mice have similar brain size and brain histology compared to their wildtype littermates. (A) Individual brain homogenates from 3–4 months old male wildtype PPM1 + / + and PPM1F ± mice were probed by Western blotting with antibodies against murine PPM1F (upper panel) or tubulin (lower panel). (B) Body weight of mice as in (A). Shown is mean ± s.e.m; n = 5; unpaired t-test, ns: not significant. (C) Brains of mice in (A) were isolated, weighted, and photographed. Scale bar: 1 cm (D) Coronal brain sections of PPM1 + / + and PPM1F ± mice were stained with cresyl violet. Lower panels show magnifications of the indicated, boxed cortical areas.

Additional file 2: Figure S2 Provides evidence that fibroblasts isolated from ppm1f-/- mice do not exhibit altered integrin surface levels or focal adhesion protein expression and details the quality control of the polyclonal anti-mousePPM1F antiserum and the polyclonal anti pT788/pT789 integrin β1 antiserum. Figure S2: Related to Fig. 4. Fibroblasts isolated from ppm1f-/- mice do not show altered focal adhesion protein expression or increased integrin surface levels. (A) WCLs of indicated MEF cell lines were analyzed by Western blotting using the purified “in-house” generated anti-mouse PPM1F antibody. As controls, WCLs of 293 T cells transfected with the empty vector (mock), GFP (GFP) or murine PPM1F (mWT) were loaded. The antibody specifically recognizes the ~ 60 kDa mouse PPM1F, but also cross-reacts with the ~ 55 kDa human PPM1F with lower specificity. Human PPM1F is endogenously expressed in HEK cells (first three samples) and re-expressed in its wildtype and phosphatase-dead form in the MEF PPM1F -/- cells (last two samples). In addition to PPM1F, a non-specific band at ~ 70 kDa is detected (*). (B) Lysates from mouse embryonic fibroblasts isolated from PPM1F + / + or littermate PPM1F-/- embryos at E10.5. PPM1F-/- cells were stably transduced with wildtype human PPM1F (hWT) or human PPM1F D360A (hDA). WCLs were subjected to Western blotting with the indicated antibodies against a panel of focal adhesion proteins; monoclonal α-tubulin antibody was used as loading control. (C) MEF cell lines from (B) were analyzed by flow cytometry for integrin surface expression levels. Cells were stained with the indicated integrin-specific antibodies. Unstained wildtype MEFs or MEFs stained with an isotype-matched irrelevant antibody (IgG control) served as controls; count ≥ 10 000 cells. (D) MEF cells were stained for the indicated integrin subunits with integrin-specific antibodies and analyzed by flow cytometry for surface expression levels; count ≥ 10 000 cells. Mean fluorescence intensity derived from one representative experiment is shown (MFI). (E) 2 µg GST (control) or GST-integrin β1 cytoplasmic domain in its wildtype, single-T788D or double-T788D/T789D mutated form were incubated with or without recombinant CaMKIIβ (120 ng) in the presence of ATP/calmodulin/CaCl2 at 30 °C for 60 min. Reactions were stopped via addition of SDS sample buffer and subjected to Western blotting using the indicated antibodies and Coomassie staining.

Additional file 3: Figure S3 shows that SK-N-MC cells silenced for PPM1F or filaminA by shRNA do not exhibit increased integrin surface levels or altered focal adhesion protein expression. Figure S3: Related to Fig. 5: PPM1F or filaminA knock-down SKN-MC cells do not show altered focal adhesion protein expression or increased integrin surface levels. (A) Whole-cell-lysates of SK-N-MC cells and SK-N-MC cells receiving shPPM1F or shfilaminA as well as SK-N-MC cells transduced with scrambled shRNA (Control) were subjected to Western blotting with the indicated antibodies against a panel of focal adhesion proteins; monoclonal α-tubulin antibody was used as loading control. (B) Bar graphs show the densitometric quantification of band intensities from PPM1F versus tubulin or from filaminA versus tubulin antibody signal from three independent experiments to evaluate the efficiency of the shRNA-mediated knock-down in SK-N-MC cells; expression in wildtype cells was set to 100%. (C) Indicated SK-N-MC cell lines were analyzed by flow cytometry for integrin surface expression levels. Cells were stained with the indicated antibodies directed against specific α or β subunits. Unstained wildtype SK-N-MC cells or cells stained with an isotype-matched irrelevant antibody (IgG control) served as controls; count ≥ 10 000 cells.

Additional file 4: Figure S4 shows representative western bots of integrin β1 immunoprecipitations and the corresponding four repetitions of the analysis of integrin β1 phosphorlyation in integrin β1 immunoprecipitates generated from PPM1F WT and PPM1F KO cells. To illustrate surface exposed integrins, cells were biotinylated and streptactin pulldown were performed. Furthermore, integrin β1 immunoprecipitates from PPM1F KO cells were treated with EndoH to analyse N-glycan composition. Figure S4: Related to Fig. 7. (A) Integrin β1 was immunoprecipitated from lysates of A172 wildtype and PPM1F KO cells using monoclonal antibody P5D2. As a control, immunoprecipitation was performed with an isotype-matched control antibody (IgG). Lysates and IPs were first probed with rabbit-monoclonal anti integrin β1 antibody D2E5. (B) Lysates as in (A) were used to immunoprecipitate integrin β1 with the mouse monoclonal antibody P5D2 (left lanes) or the rat monoclonal antibodies AIIB2 and 9EG7 (right lanes). Western Blot was performed with the polyclonal phospho-specific antibody against pT788/pT789 integrin β1 (upper panel) and upon stripping of the membrane against integrin β1 (lower panel). The bar graph depicts the densitometric evaluation pT788/pT789 signals vs. total integrin β1 observed in five repetitions of this experiment. (C) A172 wildtype and PPM1F KO cells (clone 1) were surface biotinylated and following lysis, biotinylated proteins were collected by streptavidin beads. The lysates containing biotinylated proteins and the streptavidin pull-downs (strep.) were probed as in (B) with antibodies against pT788/pT789 integrin β1 (upper panel) and against integrin β1 (lower panel). (D) Integrin β1 was immunoprecipitated from lysates of A172 wildtype and PPM1F KO cells using monoclonal antibody P5D2. As a control, immunoprecipitation was performed with an isotype-matched control antibody (IgG). Lysates and IPs were first probed with the polyclonal phospho-specific antibody against pT788/pT789 integrin β1 (upper panel) before stripping and reprobing with rabbit-monoclonal anti integrin β1 antibody D2E5 (lower panel). Shown are four repetitions of the experiment depicted also in (B). (E) Integrin β1 immunoprecipitates from lysates of PPM1F KO cells as in (A) were treated in vitro with Endoglycosidase H (EndoH) for 1 h before Western Blotting with rabbit-monoclonal anti integrin β1 antibody D2E5. Arrowheads point to the slow migrating integrin β1 band not modified by EndoH, and the faster migrating band, which has a lowered apparent molecular weight and increased mobility upon EndoH treatment.

Additional file 5: Figure S5 shows the level of re-expression of PPM1F WT and PPM1F DA mutant in PPM1F-deficient A172 cells and the resulting effect on cellular levels of pT788/pT789 integrin β1. Furthermore, the re-introduction of PPM1F into PPM1F-deficient A172 cells does not alter cell growth. Figure S5: Related to Fig. 7 and Fig. 9. (A) WCLs from A172 wildtype cells, control cells (sgRNA against Cerulean), PPM1F KO cells (sgRNA against Cerulean and PPM1F) and PPM1F KO cells re-expressing mKate2, re-expressing active PPM1F-mKate2, or re-expressing the inactive PPM1FD360A-mKate2 were analyzed by Western blotting with α-human PPM1F or α-integrin β1 antibodies. α-Tubulin antibody was used as loading control. (B) Serum-starvedA172 cell lines as in (A) were seeded onto 2 µg/ml FNIII9-11 for 45 min and WCLs were subjected to Western blotting with indicated antibodies to analyze T788/T789 phosphorylation of integrin β1. α-Tubulin antibody was used as loading control. (C) Cell growth is not altered by PPM1F re-expression. 5 × 103 A172 wildtype, PPM1F KO and PPM1F KO cells re-expressing wildtype PPM1F were seeded into 96-well plates in triplicate and examined for cell proliferation after one, two, three, and four days by paraformaldehyde fixation, crystal violet staining, dye elution with 10% acetic acid and absorbance measurement at 590 nm in a microplate reader. Shown are mean ± SEM values from one representative experiment repeated twice.

Additional file 6: Movie 1. Movie 1 shows time lapse video microscopy of serum-starved A172 wildtype cells spreading on a surface coated with 2 µg/ml FNIII9-11. Holotomographic images were taken every 4 min for 2 h 16 min starting 30 min after cell seeding. A172 wildtype cells spread fast and start to migrate. Time stamp format is hh:mm.

Additional file 7: Movie 2. Movie 2 shows time lapse video microscopy of serum-starved A172 PPM1F KO cells spreading on a surface coated with 2 µg/ml FNIII9-11. Holotomographic images were taken every 4 min for 2 h 16 min starting 30 min after cell seeding. Cells are impaired in their spreading ability despite highly dynamic actin reorganization and membrane ruffling Time stamp format is hh:mm.

Additional file 8: This additional File holds the raw images of all Western Blots and agarose gels depicted in the Main Figures and the Additional Files.

## Data Availability

All data generated or analyzed during this study are included in this published article and its supplementary information files.

## Abbreviations

BioGPS: Bio-Gene portal system
BSA: Bovine serum albumin
CAM: Chorioallantoic membrane
CamKII: Calmodulin-dependent kinase
CamKP: Calmodulin-dependent kinase phosphatase
CS: Calf serum
Dok1: Docking protein 1
E10.5: Embryonic day
EDD: Embryonic development day
EndoH: EndoglycosidaseH
FAK: Focal adhesion kinase
FANTOM5: Functional Annotation of Mammalian Genome 5
FN: Fibronectin
GFP: Green fluorescent protein
GST: Glutathion-S-transferase
GTEX: Genotype-Tissue Expression project
hDA: Human D360A mutant
HPA: Human Protein Atlas
IA: Integrin activity assay
ICAP-1: Integrin cytoplasmic domain– associated protein-1
IF: Immunofluorescence
IHC: Immunohistochemistry
ILK: Integrin-linked kinase
IP: Immunoprecipitation
MEF: Mouse embryonic fibroblast
NEAA: Non-essential amino acids
P: Phospho
P95PKL: Paxillin-kinase linker
PAK: P21-activated kinase
PBMC: Peripheral Blood Mononuclear Cell
PFA: Paraformaldehyde
PH: Periventricular heterotopia
PIX: PAK-interacting exchange factor
POPX2: Partner of PIX 2
PP2 C: Protein Phosphatase 2C
PPM: Mg2 + /Mn2 + -dependent protein phosphatase
PTPM: Protein-coding transcripts per million
RT: Room temperature
sgRNA: Single guide RNA
shRNA: Small hairpin RNA
siRNA: Small interfering RNA
TCR: T-cell-receptor
VN: Vitronectin
WB: Western Blot
X-gal: 5-Bromo-4-chloro-3-indolyl-β-D-galactopyranoside
