# Supplementary figures and images for: The phosphatase PPM1F, a negative regulator of integrin activity, is essential for embryonic development and controls tumor cell invasion
Source: BMC Biol. 2025 Jun 19;23:166. doi: 10.1186/s12915-025-02254-3 (PMC12180154; doi:10.1186/s12915-025-02254-3)

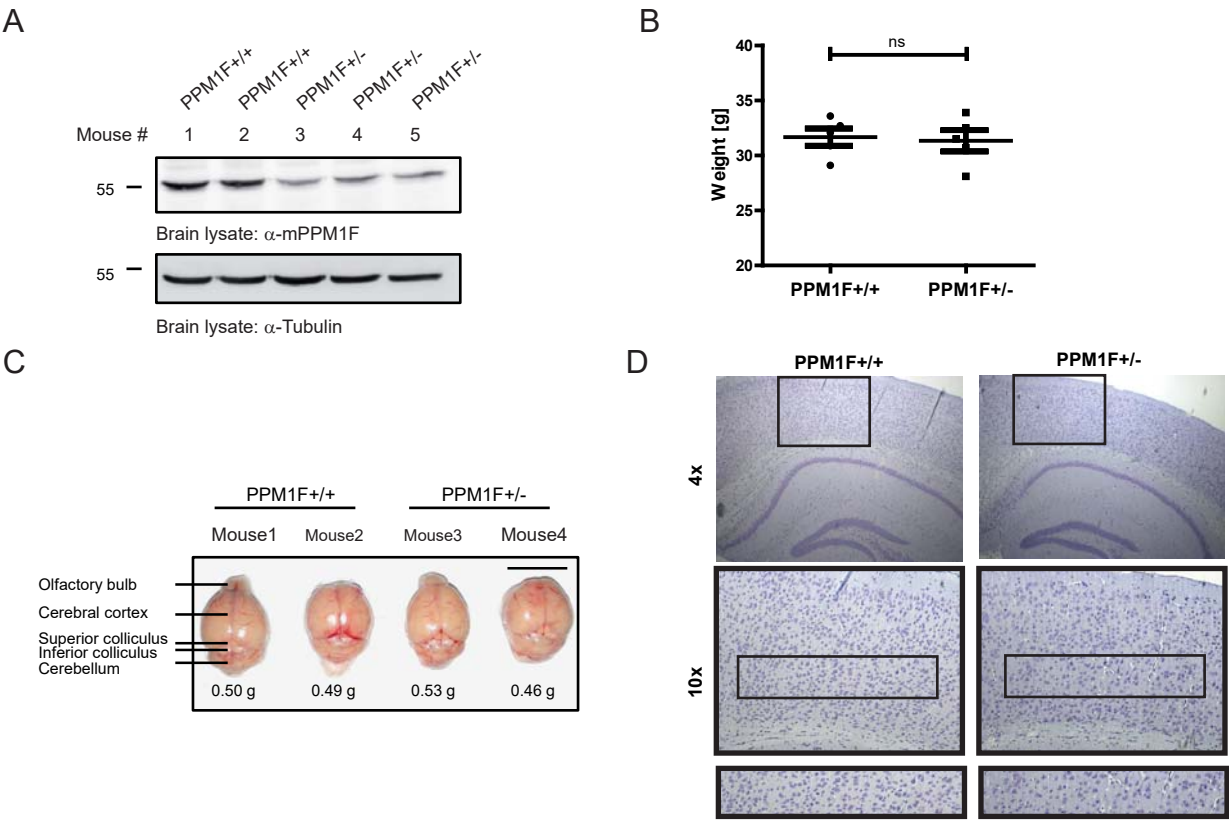

Supplement: Supplementary file 1 — Additional file 1: Figure S1 indicates similar size and brain histology of PPM1F ± mice compared to their wildtype littermates. Figure S1: Related to Fig. 3. Ppm1f ± adult mice have similar brain size and brain histology compared to their wildtype littermates. (A) Individual brain homogenates from 3–4 months old male wildtype PPM1 + / + and PPM1F ± mice were probed by Western blotting with antibodies against murine PPM1F (upper panel) or tubulin (lower panel). (B) Body weight of mice as in (A). Shown is mean ± s.e.m; n = 5; unpaired t-test, ns: not significant. (C) Brains of mice in (A) were isolated, weighted, and photographed. Scale bar: 1 cm (D) Coronal brain sections of PPM1 + / + and PPM1F ± mice were stained with cresyl violet. Lower panels show magnifications of the indicated, boxed cortical areas. [file 12915_2025_2254_MOESM1_ESM.pdf]

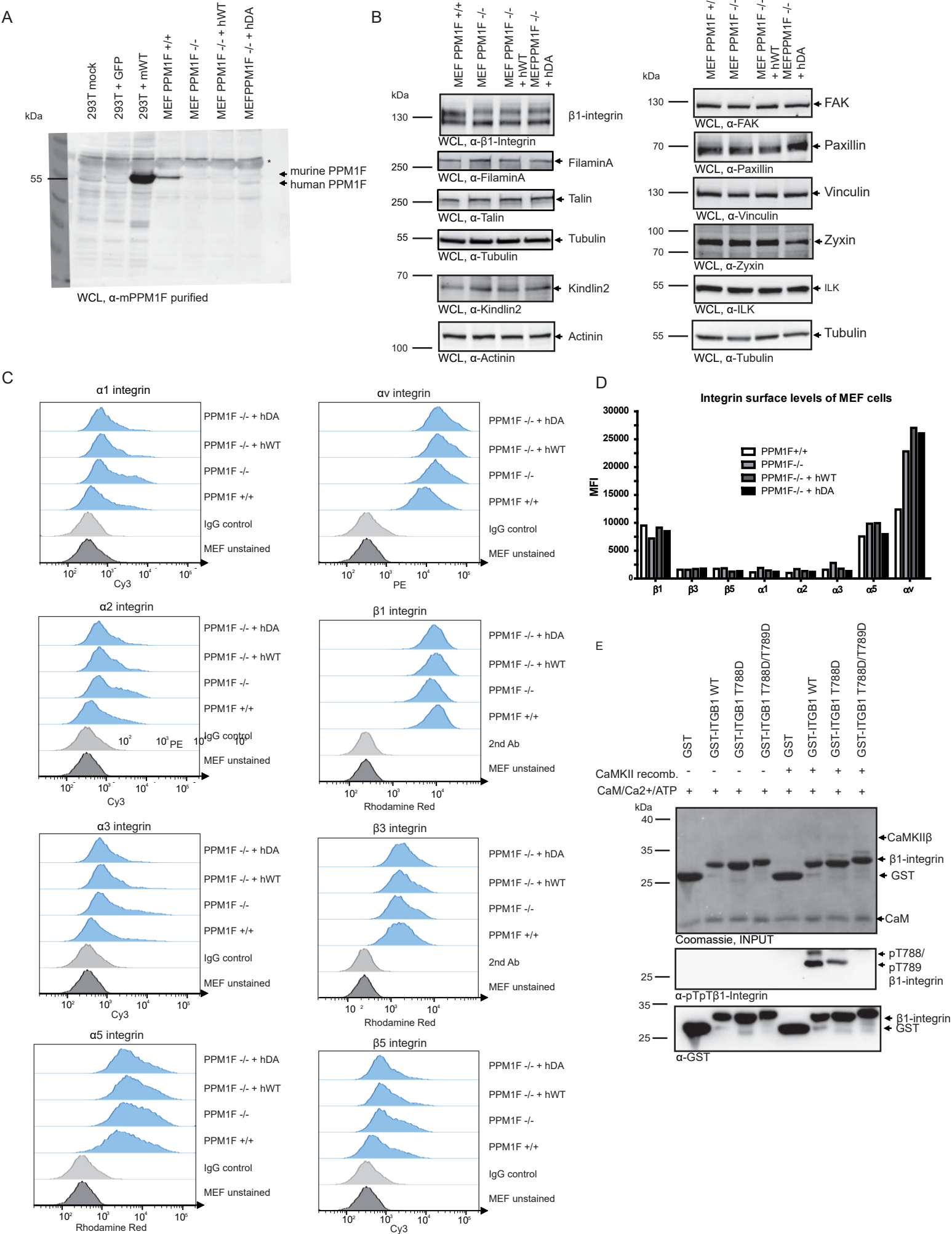

Supplement: Supplementary file 2 — Additional file 2: Figure S2 Provides evidence that fibroblasts isolated from ppm1f-/- mice do not exhibit altered integrin surface levels or focal adhesion protein expression and details the quality control of the polyclonal anti-mousePPM1F antiserum and the polyclonal anti pT788/pT789 integrin β1 antiserum. Figure S2: Related to Fig. 4. Fibroblasts isolated from ppm1f-/- mice do not show altered focal adhesion protein expression or increased integrin surface levels. (A) WCLs of indicated MEF cell lines were analyzed by Western blotting using the purified “in-house” generated anti-mouse PPM1F antibody. As controls, WCLs of 293 T cells transfected with the empty vector (mock), GFP (GFP) or murine PPM1F (mWT) were loaded. The antibody specifically recognizes the ~ 60 kDa mouse PPM1F, but also cross-reacts with the ~ 55 kDa human PPM1F with lower specificity. Human PPM1F is endogenously expressed in HEK cells (first three samples) and re-expressed in its wildtype and phosphatase-dead form in the MEF PPM1F -/- cells (last two samples). In addition to PPM1F, a non-specific band at ~ 70 kDa is detected (*). (B) Lysates from mouse embryonic fibroblasts isolated from PPM1F + / + or littermate PPM1F-/- embryos at E10.5. PPM1F-/- cells were stably transduced with wildtype human PPM1F (hWT) or human PPM1F D360A (hDA). WCLs were subjected to Western blotting with the indicated antibodies against a panel of focal adhesion proteins; monoclonal α-tubulin antibody was used as loading control. (C) MEF cell lines from (B) were analyzed by flow cytometry for integrin surface expression levels. Cells were stained with the indicated integrin-specific antibodies. Unstained wildtype MEFs or MEFs stained with an isotype-matched irrelevant antibody (IgG control) served as controls; count ≥ 10 000 cells. (D) MEF cells were stained for the indicated integrin subunits with integrin-specific antibodies and analyzed by flow cytometry for surface expression levels; count ≥ 10 000 cells. Mean flu [file 12915_2025_2254_MOESM2_ESM.pdf]

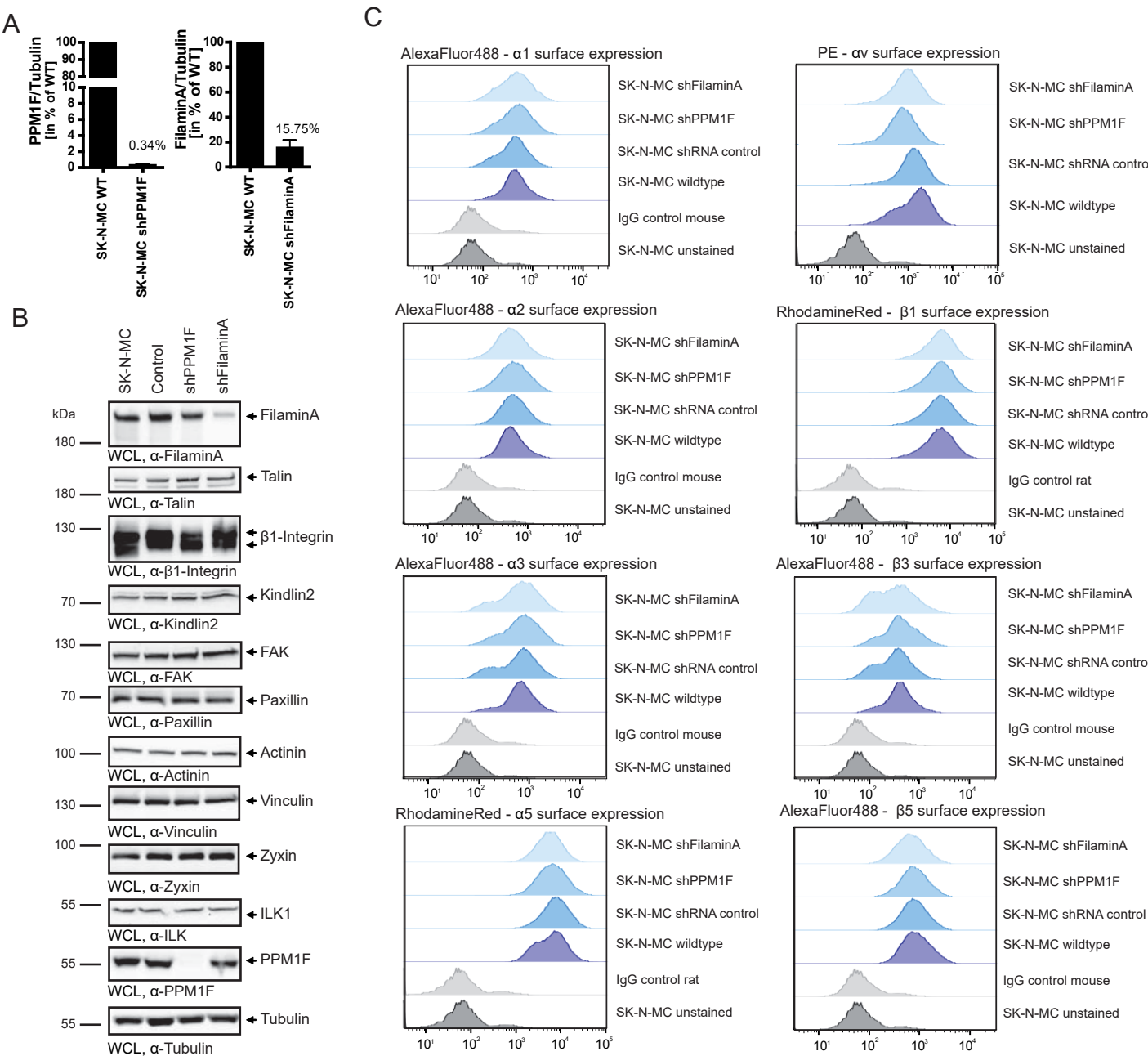

Supplement: Supplementary file 3 — Additional file 3: Figure S3 shows that SK-N-MC cells silenced for PPM1F or filaminA by shRNA do not exhibit increased integrin surface levels or altered focal adhesion protein expression. Figure S3: Related to Fig. 5: PPM1F or filaminA knock-down SKN-MC cells do not show altered focal adhesion protein expression or increased integrin surface levels. (A) Whole-cell-lysates of SK-N-MC cells and SK-N-MC cells receiving shPPM1F or shfilaminA as well as SK-N-MC cells transduced with scrambled shRNA (Control) were subjected to Western blotting with the indicated antibodies against a panel of focal adhesion proteins; monoclonal α-tubulin antibody was used as loading control. (B) Bar graphs show the densitometric quantification of band intensities from PPM1F versus tubulin or from filaminA versus tubulin antibody signal from three independent experiments to evaluate the efficiency of the shRNA-mediated knock-down in SK-N-MC cells; expression in wildtype cells was set to 100%. (C) Indicated SK-N-MC cell lines were analyzed by flow cytometry for integrin surface expression levels. Cells were stained with the indicated antibodies directed against specific α or β subunits. Unstained wildtype SK-N-MC cells or cells stained with an isotype-matched irrelevant antibody (IgG control) served as controls; count ≥ 10 000 cells. [file 12915_2025_2254_MOESM3_ESM.pdf]

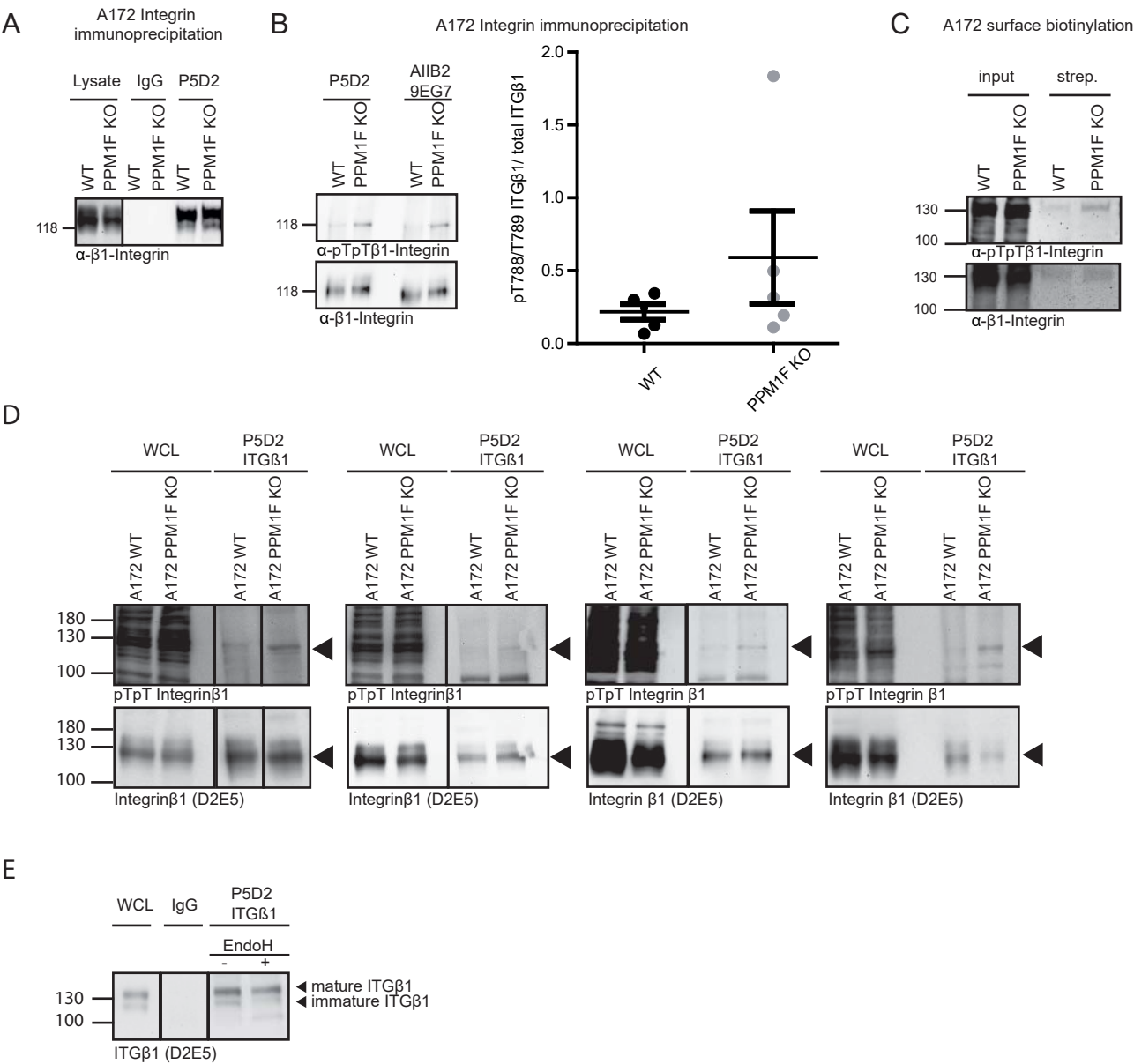

Supplement: Supplementary file 4 — Additional file 4: Figure S4 shows representative western bots of integrin β1 immunoprecipitations and the corresponding four repetitions of the analysis of integrin β1 phosphorlyation in integrin β1 immunoprecipitates generated from PPM1F WT and PPM1F KO cells. To illustrate surface exposed integrins, cells were biotinylated and streptactin pulldown were performed. Furthermore, integrin β1 immunoprecipitates from PPM1F KO cells were treated with EndoH to analyse N-glycan composition. Figure S4: Related to Fig. 7. (A) Integrin β1 was immunoprecipitated from lysates of A172 wildtype and PPM1F KO cells using monoclonal antibody P5D2. As a control, immunoprecipitation was performed with an isotype-matched control antibody (IgG). Lysates and IPs were first probed with rabbit-monoclonal anti integrin β1 antibody D2E5. (B) Lysates as in (A) were used to immunoprecipitate integrin β1 with the mouse monoclonal antibody P5D2 (left lanes) or the rat monoclonal antibodies AIIB2 and 9EG7 (right lanes). Western Blot was performed with the polyclonal phospho-specific antibody against pT788/pT789 integrin β1 (upper panel) and upon stripping of the membrane against integrin β1 (lower panel). The bar graph depicts the densitometric evaluation pT788/pT789 signals vs. total integrin β1 observed in five repetitions of this experiment. (C) A172 wildtype and PPM1F KO cells (clone 1) were surface biotinylated and following lysis, biotinylated proteins were collected by streptavidin beads. The lysates containing biotinylated proteins and the streptavidin pull-downs (strep.) were probed as in (B) with antibodies against pT788/pT789 integrin β1 (upper panel) and against integrin β1 (lower panel). (D) Integrin β1 was immunoprecipitated from lysates of A172 wildtype and PPM1F KO cells using monoclonal antibody P5D2. As a control, immunoprecipitation was performed with an isotype-matched control antibody (IgG). Lysates and IPs were first probed with the polyclonal phospho-specific antibody agai [file 12915_2025_2254_MOESM4_ESM.pdf]

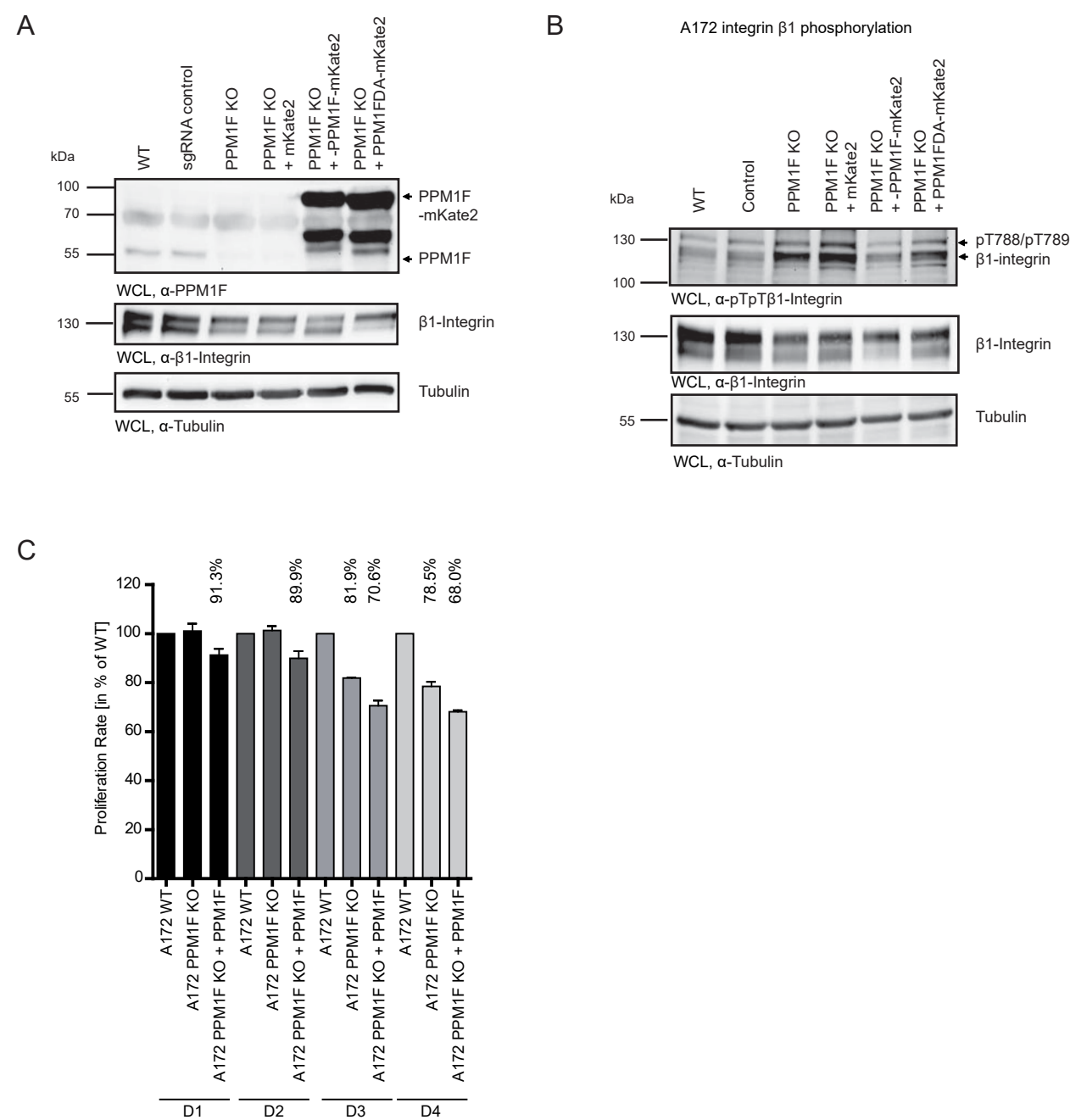

Supplement: Supplementary file 5 — Additional file 5: Figure S5 shows the level of re-expression of PPM1F WT and PPM1F DA mutant in PPM1F-deficient A172 cells and the resulting effect on cellular levels of pT788/pT789 integrin β1. Furthermore, the re-introduction of PPM1F into PPM1F-deficient A172 cells does not alter cell growth. Figure S5: Related to Fig. 7 and Fig. 9. (A) WCLs from A172 wildtype cells, control cells (sgRNA against Cerulean), PPM1F KO cells (sgRNA against Cerulean and PPM1F) and PPM1F KO cells re-expressing mKate2, re-expressing active PPM1F-mKate2, or re-expressing the inactive PPM1FD360A-mKate2 were analyzed by Western blotting with α-human PPM1F or α-integrin β1 antibodies. α-Tubulin antibody was used as loading control. (B) Serum-starvedA172 cell lines as in (A) were seeded onto 2 µg/ml FNIII9-11 for 45 min and WCLs were subjected to Western blotting with indicated antibodies to analyze T788/T789 phosphorylation of integrin β1. α-Tubulin antibody was used as loading control. (C) Cell growth is not altered by PPM1F re-expression. 5 × 10^3^ A172 wildtype, PPM1F KO and PPM1F KO cells re-expressing wildtype PPM1F were seeded into 96-well plates in triplicate and examined for cell proliferation after one, two, three, and four days by paraformaldehyde fixation, crystal violet staining, dye elution with 10% acetic acid and absorbance measurement at 590 nm in a microplate reader. Shown are mean ± SEM values from one representative experiment repeated twice. [file 12915_2025_2254_MOESM5_ESM.pdf]
