# Supplementary material for: The phosphatase PPM1F, a negative regulator of integrin activity, is essential for embryonic development and controls tumor cell invasion
Source: BMC Biol. 2025 Jun 19;23:166. doi: 10.1186/s12915-025-02254-3 (PMC12180154; doi:10.1186/s12915-025-02254-3)

Raw Blots / Figure 1

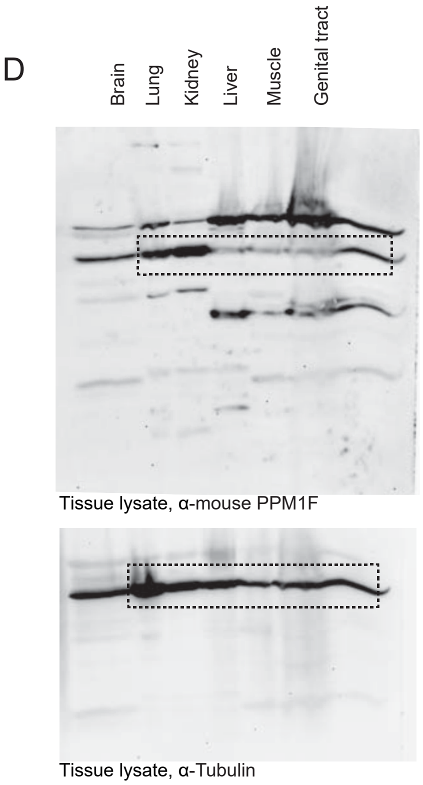

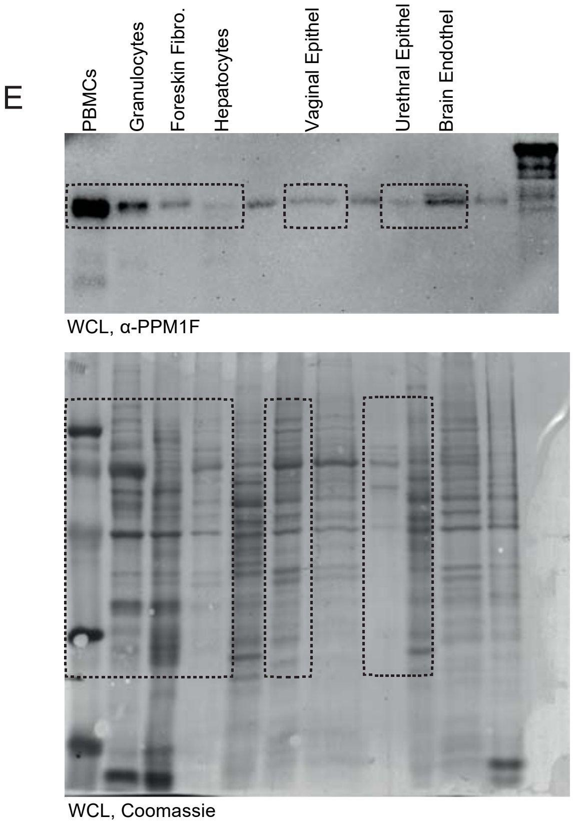

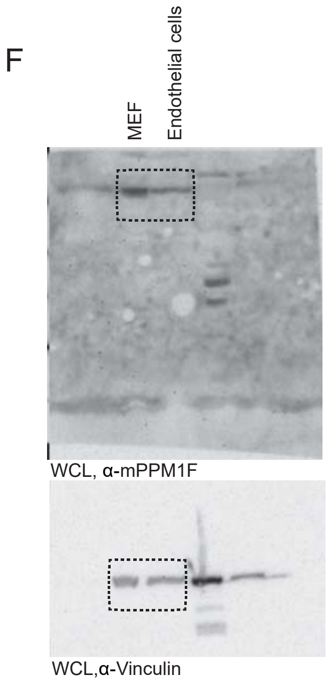

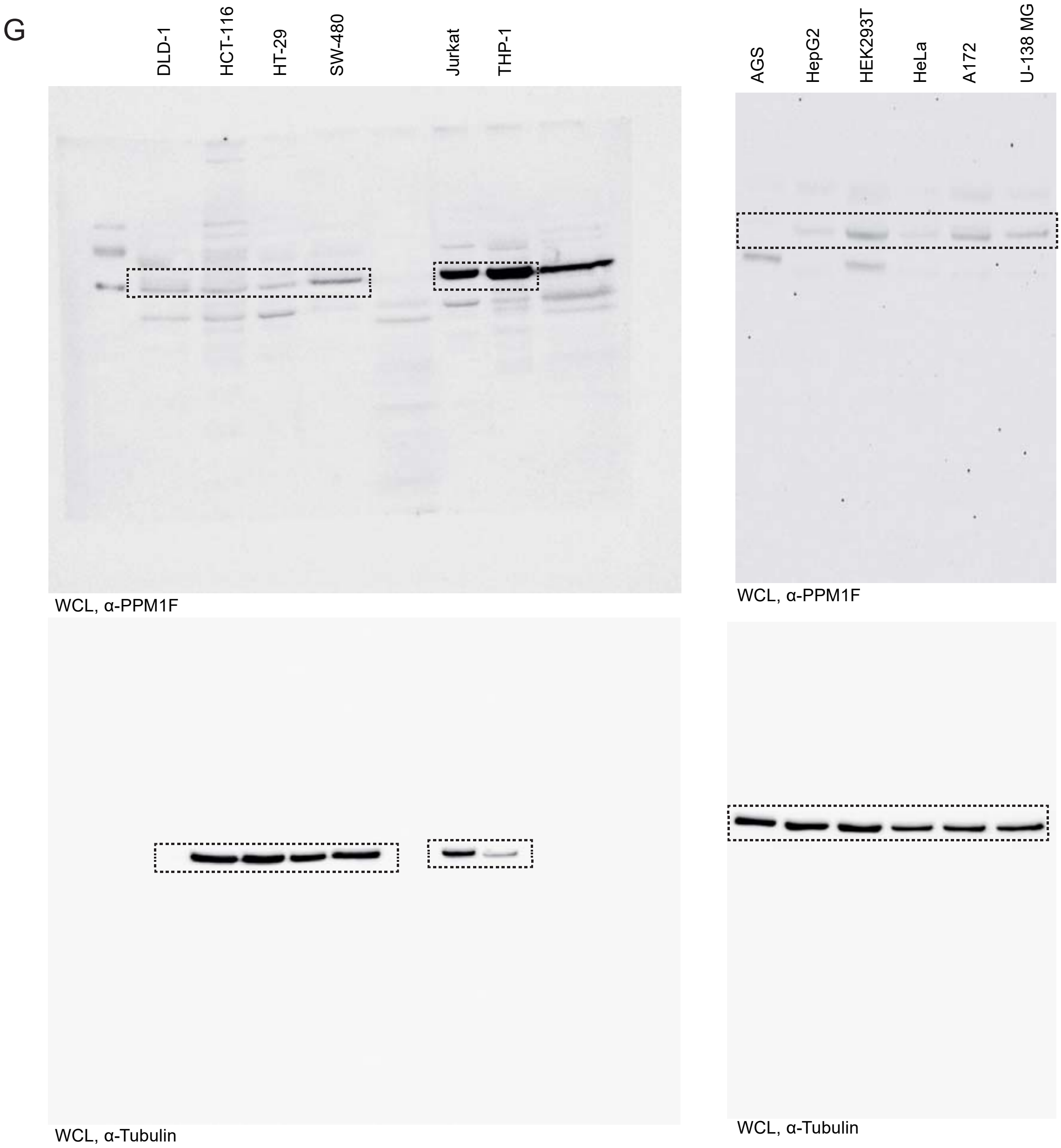

Raw Blots / Figure 2

A

Embryo1 Embryo2 Embryo3 Embryo4 Embryo5 Embryo6

wt tg wt tg wt tg wt tg wt tg wt tg

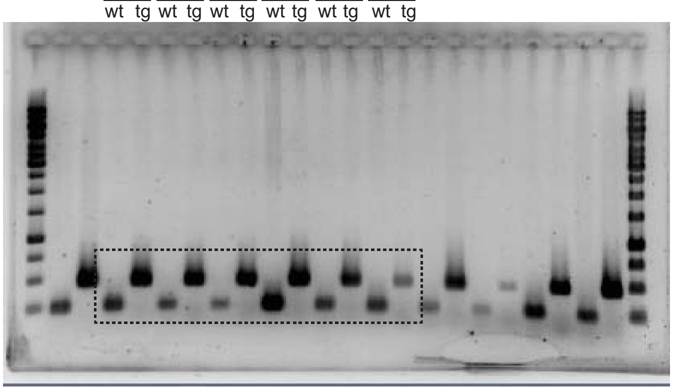

E12.5

Tail biopsies

Embryo1 Embryo2 Embryo3 Embryo4 Embryo5 Embryo6

wt tg wt tg wt tg wt tg wt tg wt tg

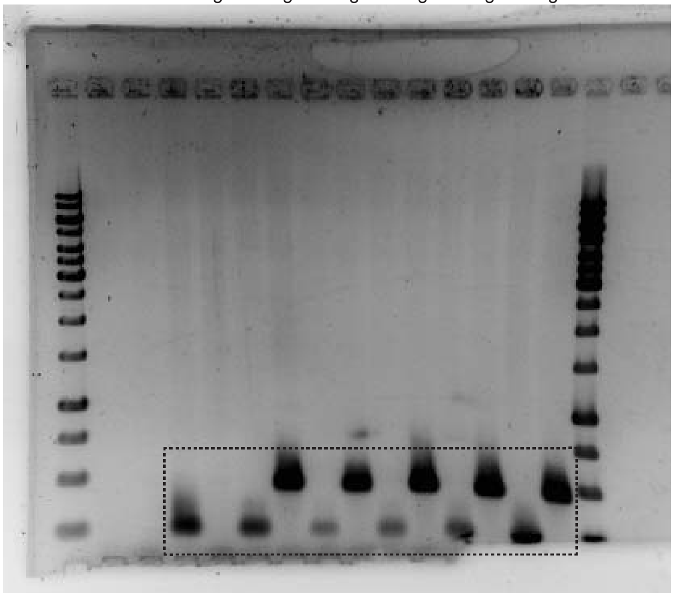

E13.5

Tail biopsies

Embryo1 Embryo2 Embryo3 Embryo4 Embryo5 Embryo6 Embryo7

wt tg wt tg wt tg wt tg wt tg wt tg wt tg

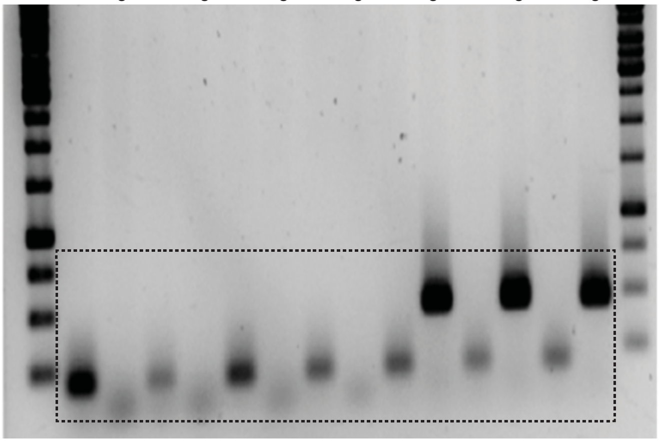

E14.5

Tail biopsies

B

Isolated MEFs

PPM1F +/+ PPM1F -/-

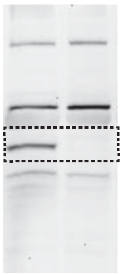

WCL, α-mPPM1F

Isolated MEFs

PPM1F +/+ PPM1F -/-

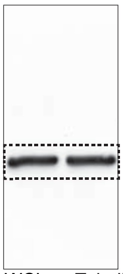

WCL, α-Tubulin

C

Isolated MEFs

PPM1F +/- PPM1F +/+ PPM1F -/-

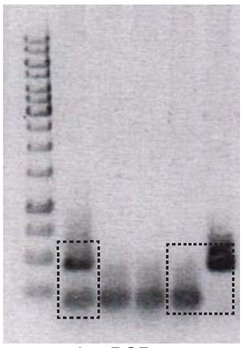

genotyping PCR

Raw Blots / Figure 4

A

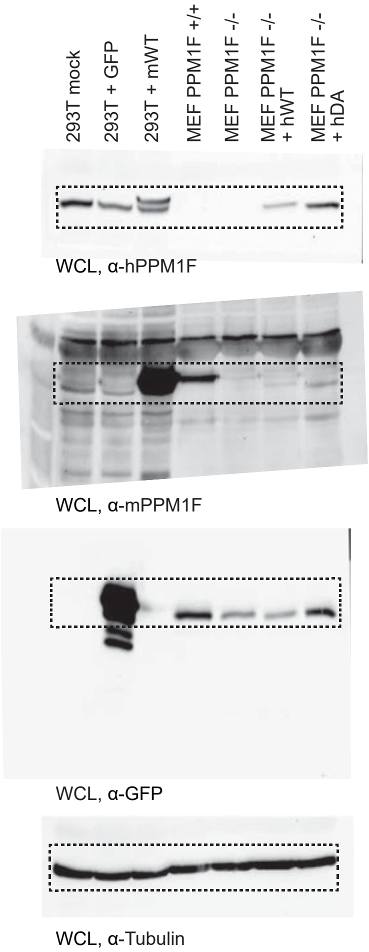

Raw Blots / Figure 5

A

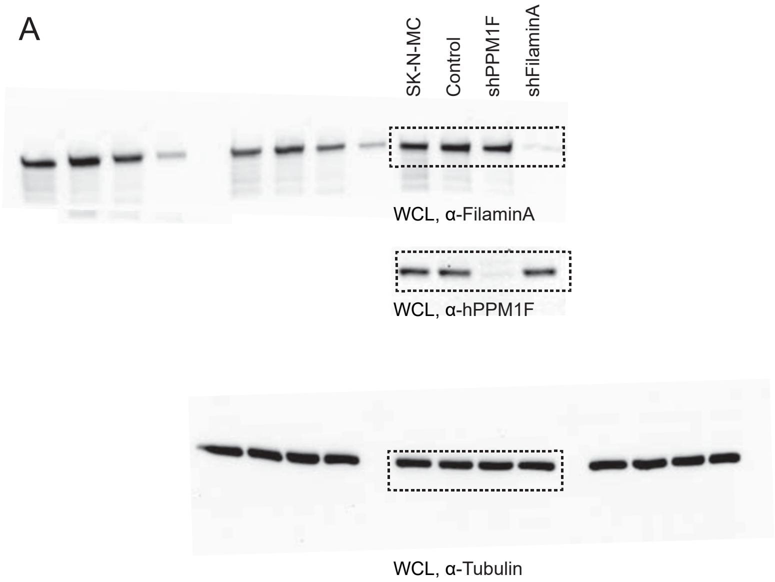

E

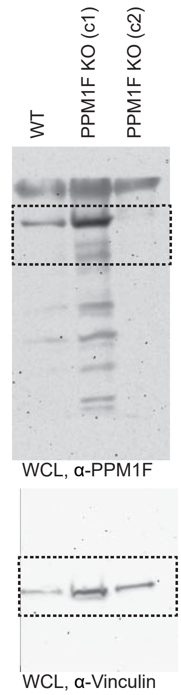

Raw Blots / Figure 6

A

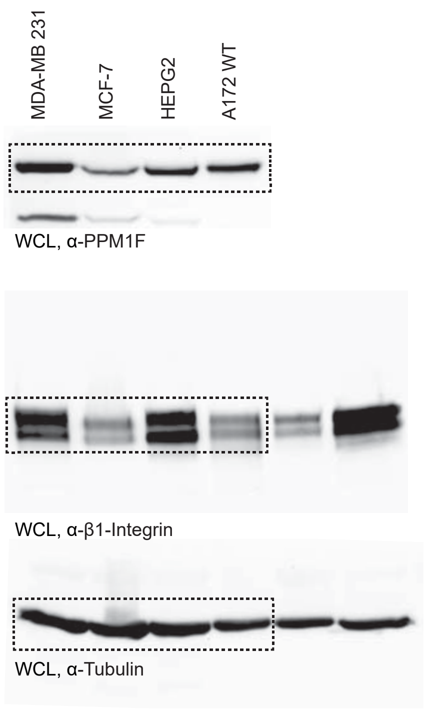

D

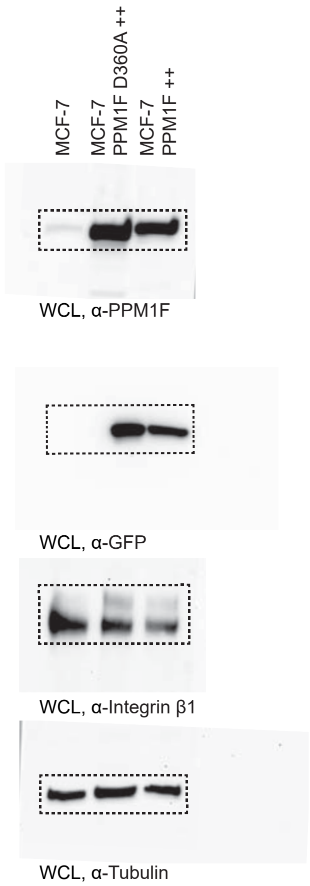

Raw Blots / Figure 7

A

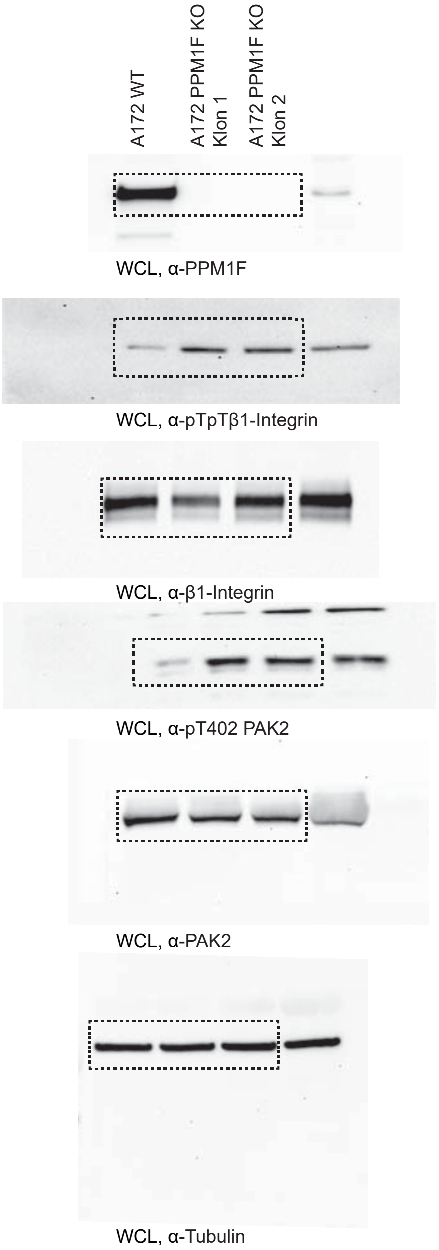

Raw Blots / Figure 8

A

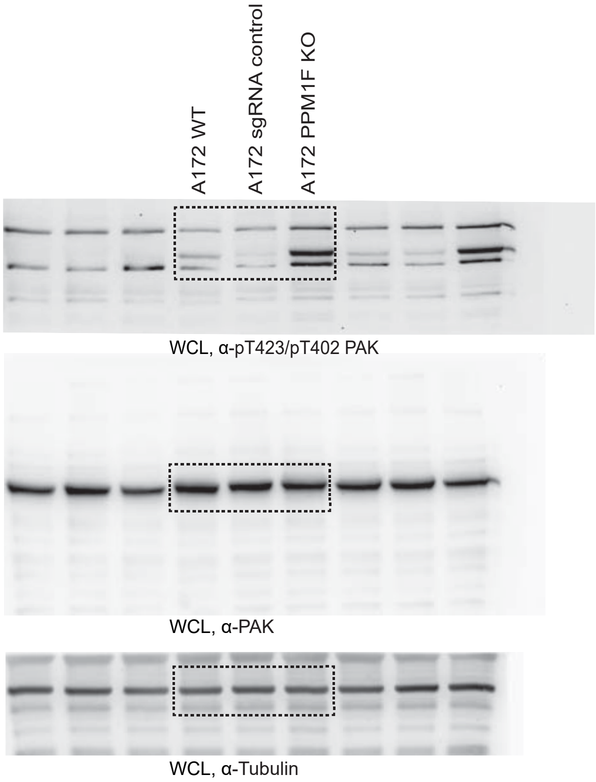

Raw Blots / Additional File 1

A

PPM1F+/+ PPM1F+/+ PPM1F+/- PPM1F+/- PPM1F+/-

Mouse # 1 2 3 4 5

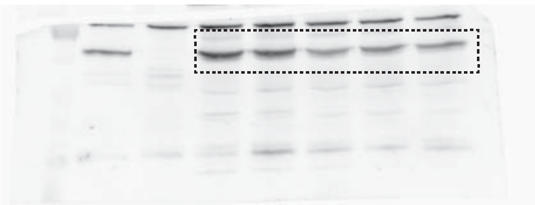

Brain lysate: a-mPPM1F

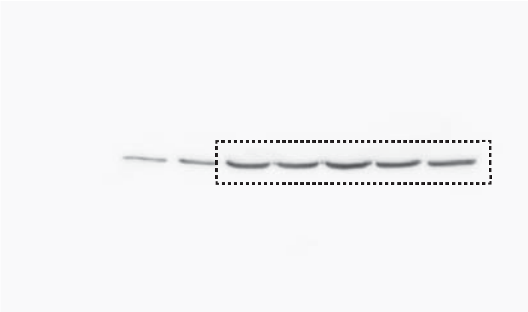

Brain lysate: a-Tubulin

Raw Blots / Additional File 2

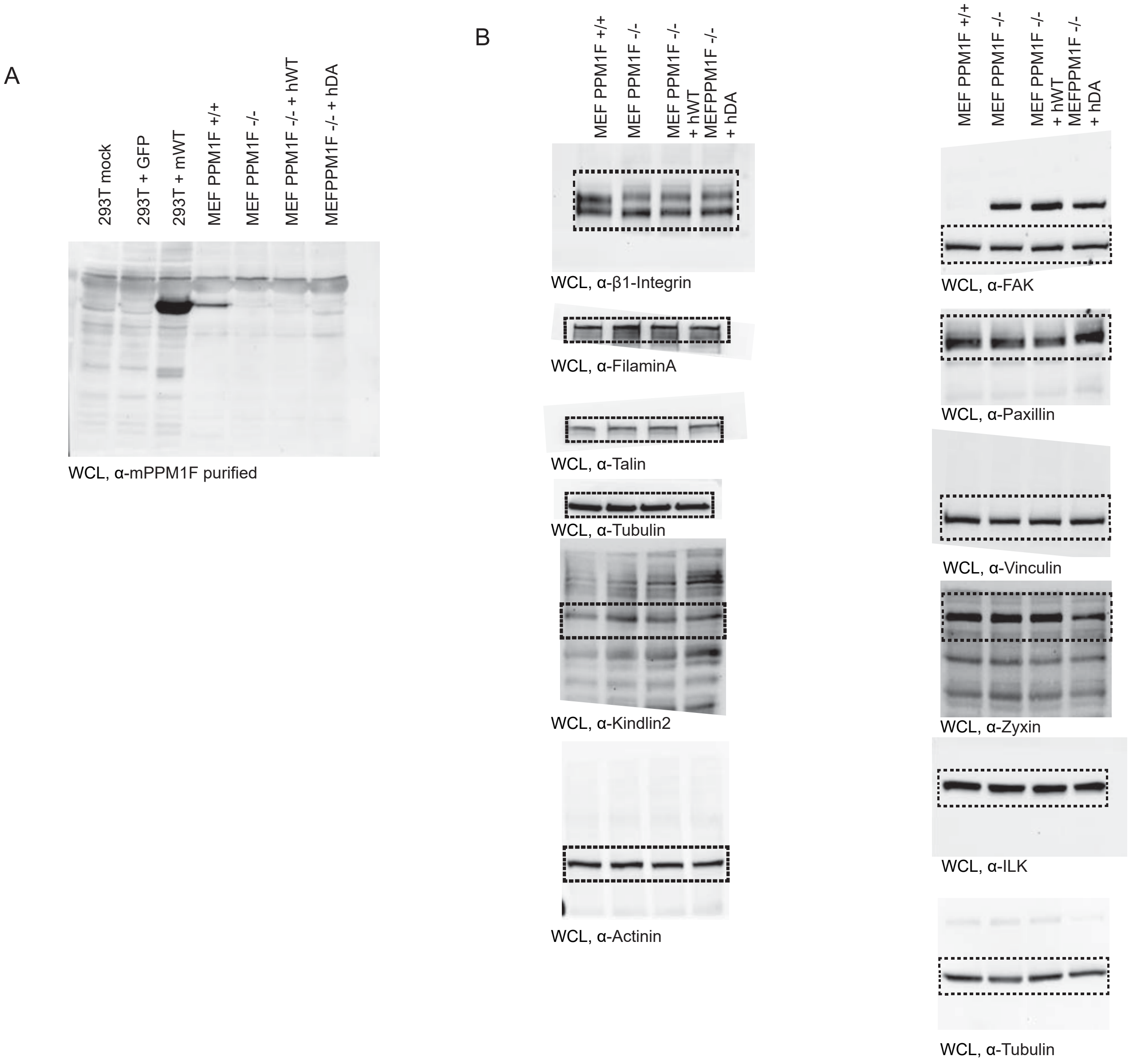

Raw Blots / Additional File 3

B

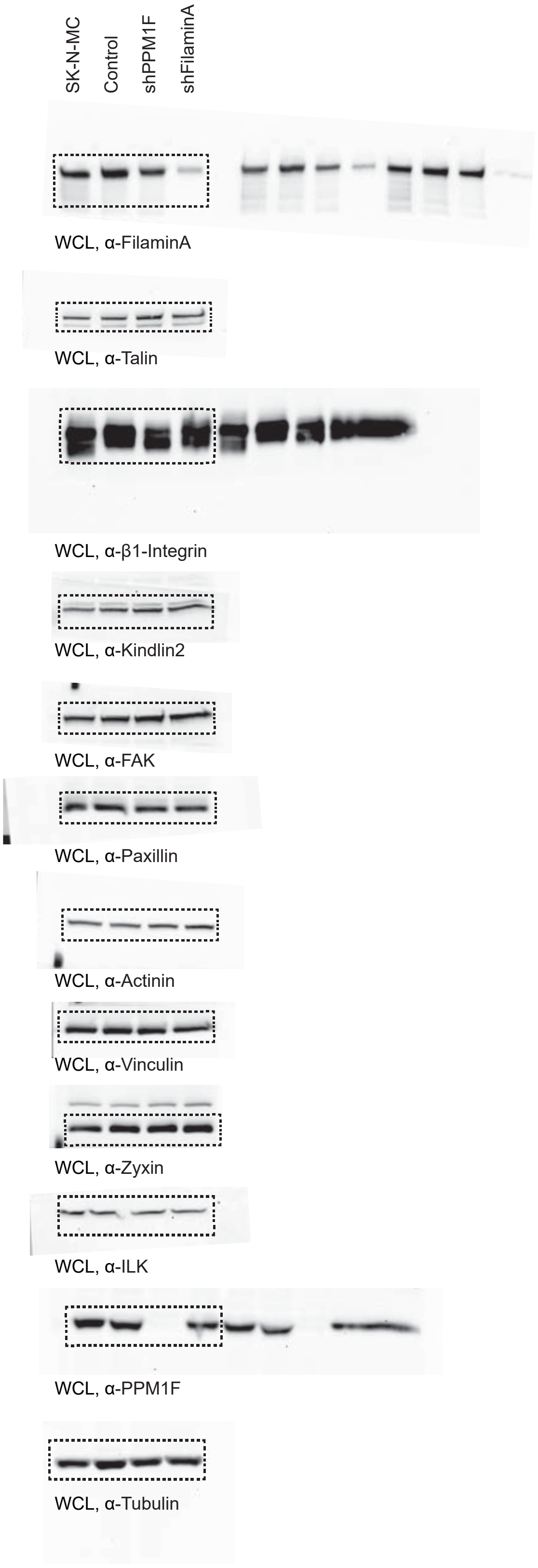

Raw Blots / Additional File 4

A

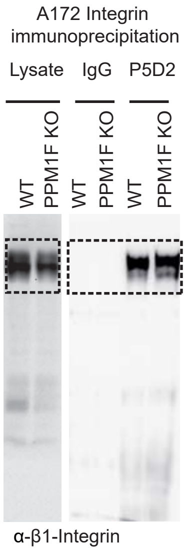

B

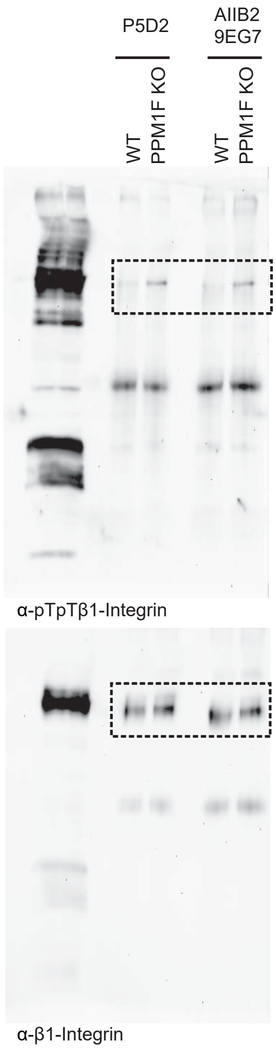

C

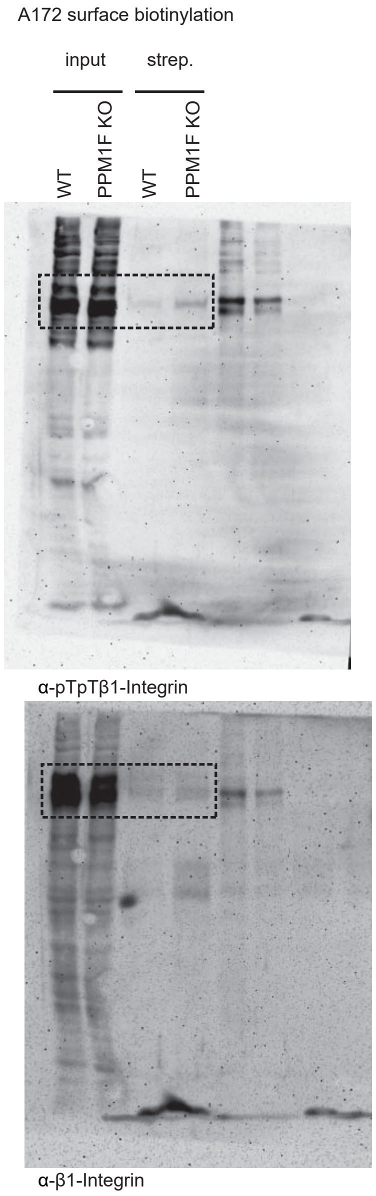

Raw Blots / Additional File 4

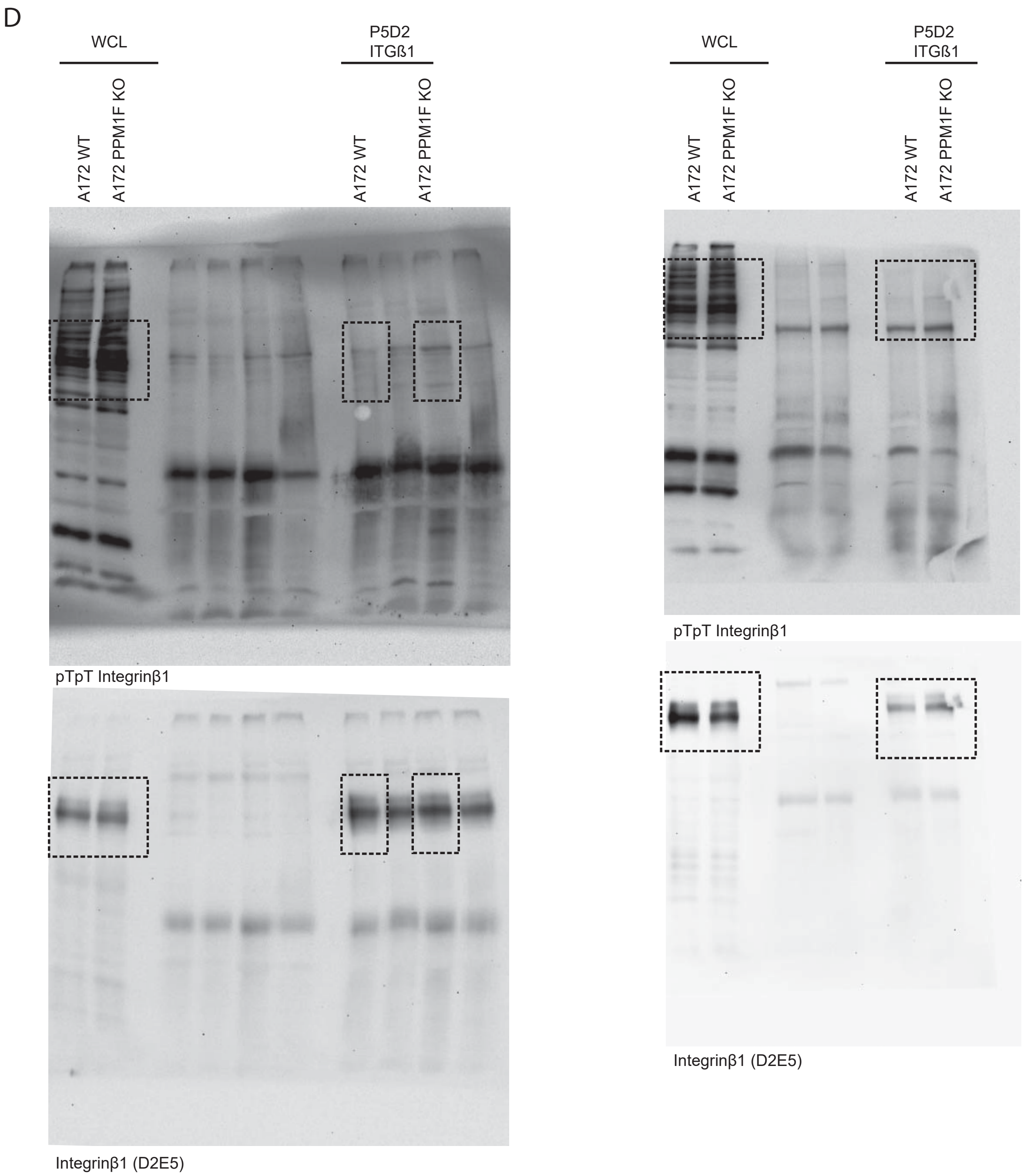

Raw Blots / Additional File 4

D

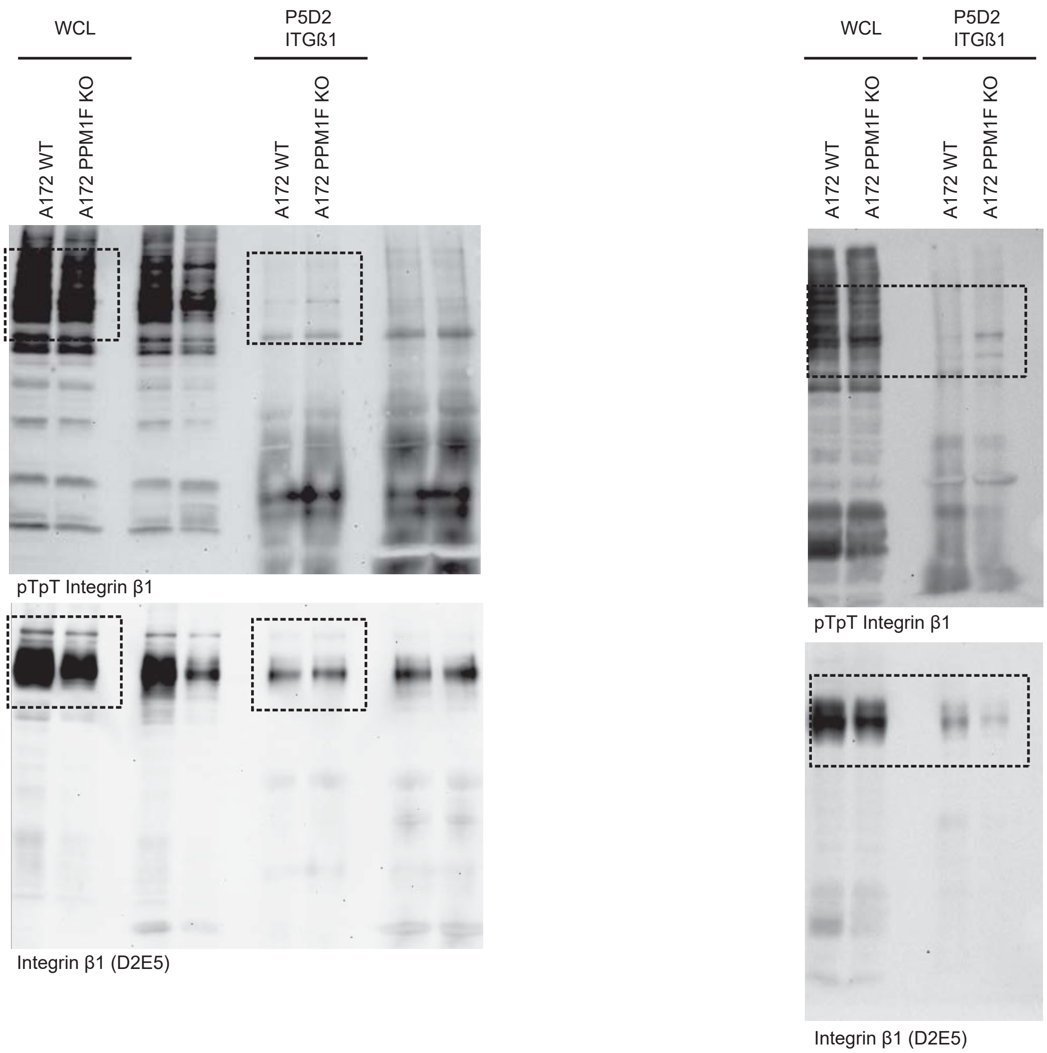

Raw Blots / Additional File 4

E

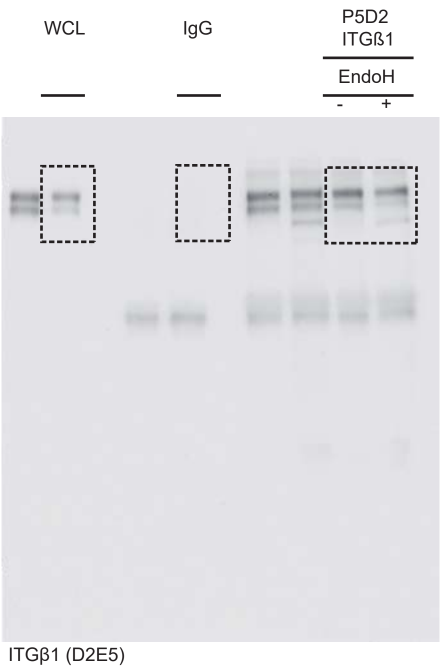

Raw Blots / Additional File 5

A

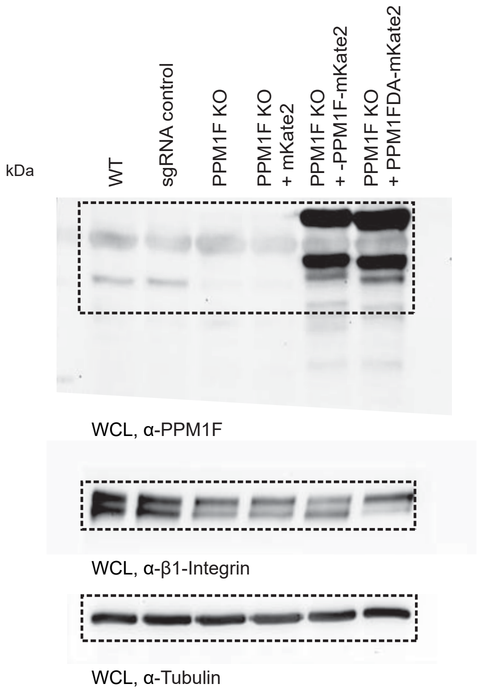

B

A172 integrin β1 phosphorylation

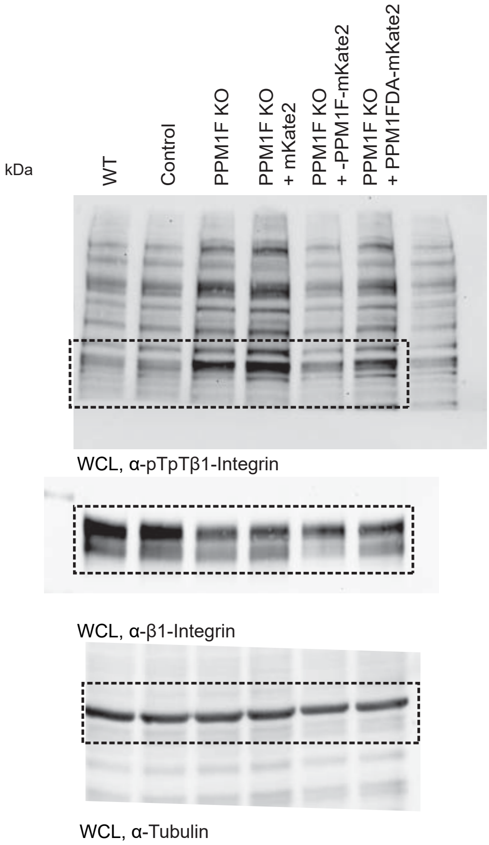

Supplement: Supplementary file 8 — Additional file 8: This additional File holds the raw images of all Western Blots and agarose gels depicted in the Main Figures and the Additional Files. [file 12915_2025_2254_MOESM8_ESM.pdf]
